# Enhancing G‐Quadruplex Binding: Rational Design and Biophysical Evaluation of Dimeric Ligands

**DOI:** 10.1002/chem.202503128

**Published:** 2025-11-23

**Authors:** Matteo Giannangeli, Nicolò Dal Ponte, Margrate Anyanwu, Martina Brigida Romanello, Ernesto Mucenji, Riccardo Rigo, Giovanni Ribaudo, Claudia Sissi, Alessandra Gianoncelli

**Affiliations:** ^1^ Department of Molecular and Translational Medicine University of Brescia Brescia Italy; ^2^ Department of Pharmaceutical and Pharmacological Sciences University of Padova Padova Italy

**Keywords:** anthracene, anthraquinone, G‐quadruplex, molecular tweezers, telomere

## Abstract

G‐quadruplexes (G4s) are non‐canonical nucleic acid structures formed by guanine‐rich sequences. Within the human genome, beyond telomeres, G4 motifs have also been found in promoters, implying a potential role in gene regulation that could be achieved through interactions with small‐molecule ligands. In this field, anthraquinone represented a privileged scaffold for the design of G4‐targeting molecules. Additionally, emerging evidence proved that compounds bearing two aromatic cores can offer enhanced affinity and selectivity toward G4s due to cooperative binding in a tweezer‐like fashion. This study reports the rational design and synthesis of anthraquinone‐based building blocks conceived to generate a dimeric ligand connected through a flexible polyethylene glycol (PEG) linker to potentially recognize G4 as molecular tweezers. The interaction of the synthesized compounds with G4 structures has been investigated by biophysical techniques, including fluorescence thermal shift assays, electrospray ionization‐mass spectrometry and circular dichroism (CD) spectroscopy, supported by molecular modeling studies. Finally, cytotoxicity assays were performed to evaluate the antiproliferative effects of the new ligands.

## Graphical abstract

Fluorescence thermal shift assays, electrospray ionization‐mass spectrometry, CD spectroscopy, molecular modeling and cytotoxicity assays were enrolled to study the binding of a rationally designed anthraquinone‐based G‐quadruplex binder interacting with the nucleic acid as a molecular tweezer.

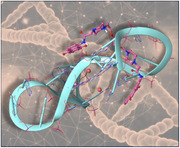

## 1 Introduction

G‐quadruplexes (G4s) are non‐canonical nucleic acid secondary structures that can arise at genomic sites highly enriched in guanines, where “G‐tetrads”—square planar arrays of four guanines that interact through Hoogsteen hydrogen bonds can form [1, 2]. The stacking of two or more G‐tetrads leads to the final G4 structure, which is further stabilized by the coordination in the central core of monovalent cations such as Na^+^ and K^+^ [3] A typical feature of G4s is their highly polymorphic nature, as they can fold into different topologies, with the DNA strands organized according to parallel, antiparallel, or hybrid relative orientation [4, 5, 6]. The preferential folding into one of them is largely dictated by the nature of the coordinated cations [3], as well as by the loop composition and length [7] that drive variable anti/syn sugar conformations [8].

Along the genome, G4s are predicted to be enriched at telomers, gene promoters, transcription starting site (TSS), 5’ untranslated region (5’UTR), and introns [9, 10]. This suggested that G4s might be exploited to regulate the expression of those genes related to cancer development [11]. Accordingly, over the years, several small molecules have been developed to selectively target these DNA arrangements [12]. The majority of the identified G4 ligands share common structural features: a flat aromatic surface(s) that stack mostly on the terminal G‐tetrads through π‐π interactions and side chains with basic functional groups to interact with the negatively charged phosphates in the backbone [13]. These features frequently allow them to exhibit a good selectivity toward G4s over double‐stranded DNAs, although they still fail to discriminate among different G4 topologies [11, 14].

Anthraquinone is a well‐known versatile scaffold for the design of DNA binders. On this core, the side chains chemical composition and position drive the preferential interactions with different nucleic acid arrangements. This led to the development of promising G4 binders that generally work as stacking agents [15, 16, 17].

To further improve G4 targeting, many studies reported the use of dimeric ligands where two aromatic cores are linked by a proper spacer [18, 19]. This design should ideally improve the binding, as originally reported by Jain et al. 2012 [20], who showed that a dimeric form of 1, 3‐phenylenebis‐benzimidazole demonstrated increased G4 binding affinity (BA), thermal stability, and telomerase inhibition activities compared to analogues with a single core. Notably, the chemical features and the length of the spacers have also proved to affect the interaction with G4 [21, 22].

To shed light on the G4 recognition feature of dimeric ligands, here we considered connecting two units of an optimized anthraquinone core through a flexible polyethylene glycol (PEG) linker. These molecules are expected to function as molecular tweezers, aiding the cooperative stacking of the two polycyclic aromatic moieties on the external G‐quartets, thereby enabling efficient G4 recognition. As DNA target model, we selected the human telomeric sequence since it folds into a highly polymorphic G4, thus it is expected to easily rearrange to properly accommodate the newly synthesized ligands.

## 2 Results and Discussion

### 2.1 Rationale of the Design, Synthesis, and Evaluation of G4 Binding Building Blocks

Ideally, through an induced‐fit recognition mechanism, flexible molecular tweezers could adopt the most appropriate conformation for binding to the nucleic acid. For G4 recognition, they are expected to show adaptability to the G4 topology, to support variable interaction modes and to generate a cooperative binding between the two aromatic cores [22]. Due to these reasons, we selected a PEG‐based spacer that, thanks to its flexibility and hydrophilicity, should allow the molecule to optimally adapt to the nucleic acid arrangements, as previously demonstrated by Maji et al. [21]. On the same basis, we add a piperazine ring between the cores and the spacer to provide a potential ionic pair with the phosphate groups.

**SCHEME 1:**
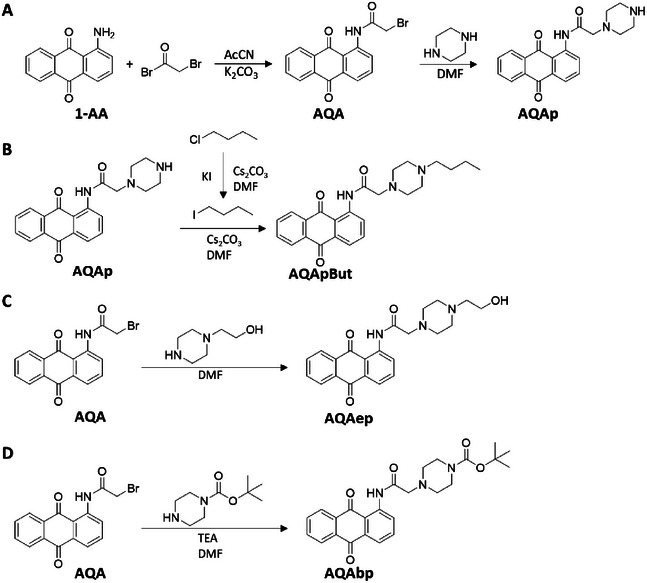
Synthetic scheme for the preparation of AQAp (A), AQApBut (B), AQAep (C), and AQAbp (D) starting from 1‐aminoanthraquinone (1‐AA).

In our study, 1‐aminoanthraquinone was selected as the starting unit for designing novel molecular tweezers. The synthetic pathways for the preparation of the derivatized anthraquinone scaffolds, which were tested to identify the best monomeric core, are reported in Scheme 1.

To obtain the first intermediates of this series, an acylation between the haloacyl bromide and 1‐aminoanthraquinone was performed. Then, the following reaction consisted in a nucleophilic substitution between the compounds obtained in the previous step and piperazine. In this phase, AQApBut and AQAep derivatives were also synthesized, with the aim of exploring the contribution of the scaffold and of different degrees of derivatization to the interaction with nucleic acids. Similarly, N‐Boc piperazine was used to obtain AQAbp and to explore the influence of a substitution on the piperazine constituted by a sp^2^ carbon atom and a bulkier group.

**FIGURE 1:**
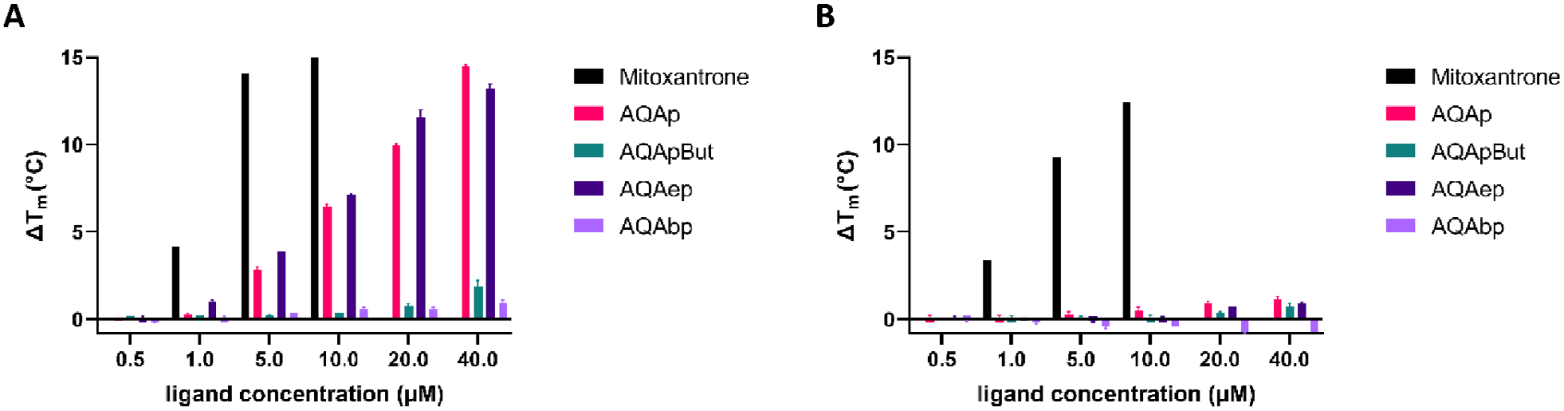
Fluorescence thermal shift experiments. Variation of the melting temperature induced by increasing concentrations of the analyzed anthraquinone derivatives on (**A**) Tel23‐FD and (**B**) scr‐dsDNA in 10 mM Li_3_(PO)_4_, 50 mM KCl, pH 7.5.

The potential interaction of this first set of synthesized derivatives with DNA was preliminarily screened by a fluorescence thermal shift assay (FTSA) using two oligonucleotide models: a telomeric sequence that folds into a single G4 (Tel23‐FD) and a double‐stranded DNA obtained by the pairing of an oligonucleotide unable to fold into G4 but with the same base composition of Tel23, thus resulting, to its complementary strand (scr‐dsDNA). As a reference compound, we included mitoxantrone (MTX), a well‐known DNA binder that shares the anthraquinone core with our compounds [23, 24]. The observed variations of the DNA melting temperature induced by increasing concentrations of the tested ligands are reported in Figure 1.

These results clearly highlighted that all the new ligands were less efficient than MTX, and only AQAp and AQAep were able to significantly stabilize the telomeric G4 in the tested concentration range. This indicated that the addition of an aliphatic side chain at the piperazine amine fully elicited G4 recognition which, conversely, was fully preserved in the presence of a hydroxy‐ethyl chain. Noteworthy, while MTX did not discriminate between the two tested DNA secondary structures, none of the new derivatives stabilized dsDNA.

These findings supported the anthraquinone scaffold of AQAp as a promising monomeric unit for our molecular tweezers.

### 2.2 Synthesis and G4 Binding Evaluation of Dimeric Binders

**SCHEME 2:**
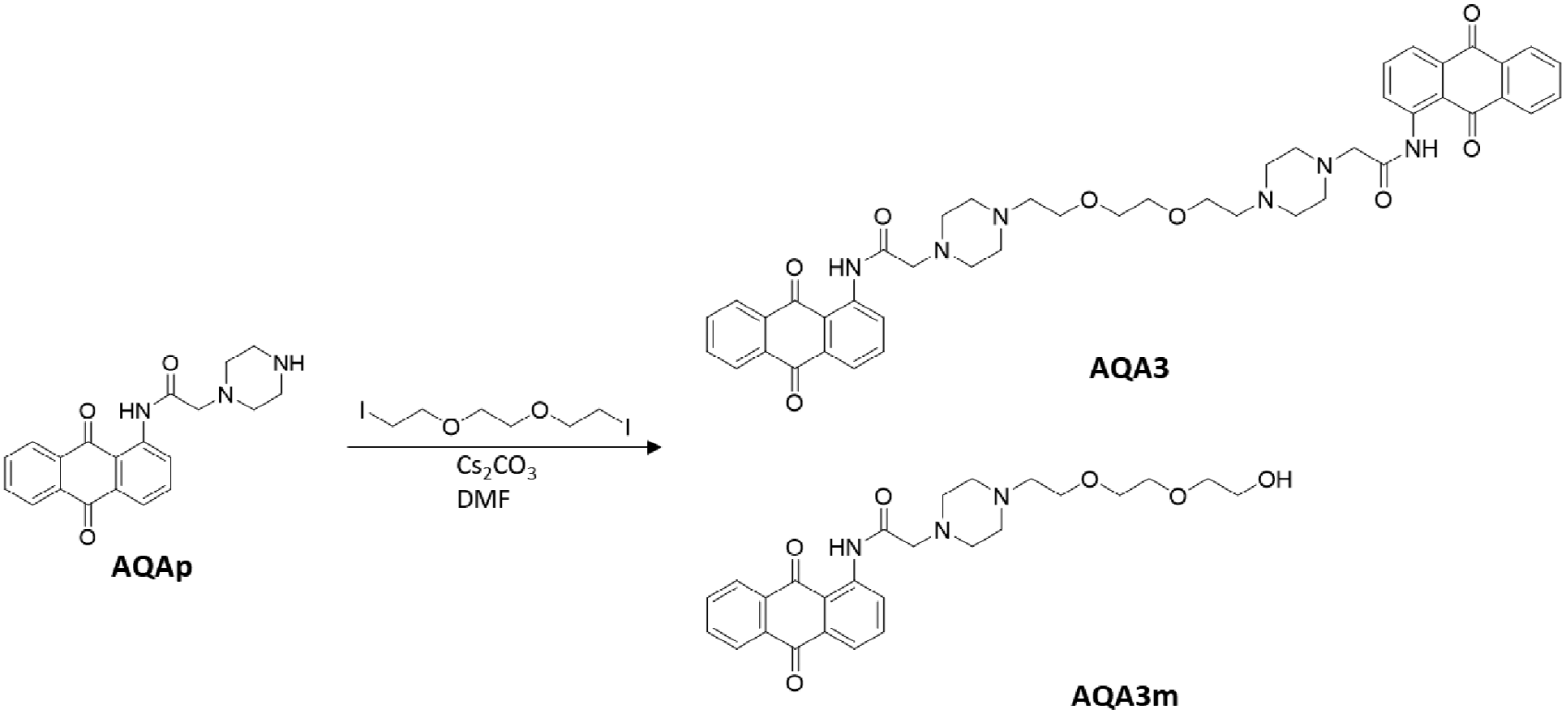
Synthetic scheme for the preparation of AQA3. As detailed in the Experimental Section, the final step allowed the isolation also of the mono‐substituted derivative, namely AQA3m.

In agreement with previously reported data on dimeric G4 binders, a 3‐unit PEG chain was selected as linker moiety [20]. The synthetic pathway for the preparation of the bifunctional compound AQA3, which also led to the isolation of AQA3m, is reported in Scheme 2. To synthesize AQA3, a nucleophilic substitution was performed between two piperazine derivatives and an activated oligo‐ethylene glycol that serves as a linker between the two aromatic scaffolds in a one‐pot procedure.

**SCHEME 3:**
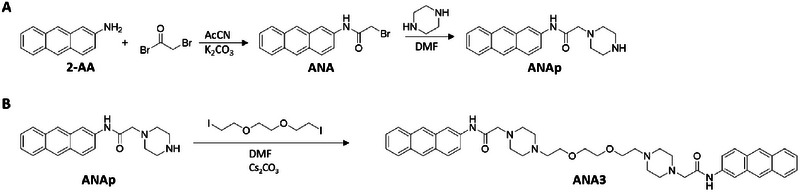
Synthetic scheme for the preparation of ANAp (A) and ANA3 (B) starting from 2‐aminoanthracene (2‐AA).

With the aim of exploring and confirming the importance of the anthraquinone scaffold for the interaction with nucleic acids, we also synthesized the analogues based on the anthracene scaffold, which is another motif known for its interaction with G4s [25]. This led to the synthesis of the monomeric ANAp and the dimeric ANA3. Through a similar synthetic approach, reported in Scheme 3, we obtained the two derivatives ANAp and ANA3.

The DNA recognition properties of these novel monomeric (AQAp and ANAp) and dimeric (AQA3 and ANA3) compounds were thus evaluated. For proper comparison, derivative AQA3m, which corresponds to the monomeric AQAp properly functionalized with the selected linker, was included. This analysis was performed by electrospray ionization‐mass spectrometry (ESI‐MS), as a valuable biophysical tool for studying DNA‐ligand interactions. Indeed, the proper tuning of instrumental parameters preserves the noncovalent complexes in the gas phase, thus allowing for the determination of BA, stoichiometry, and selectivity [26, 27, 28].

**FIGURE 2:**
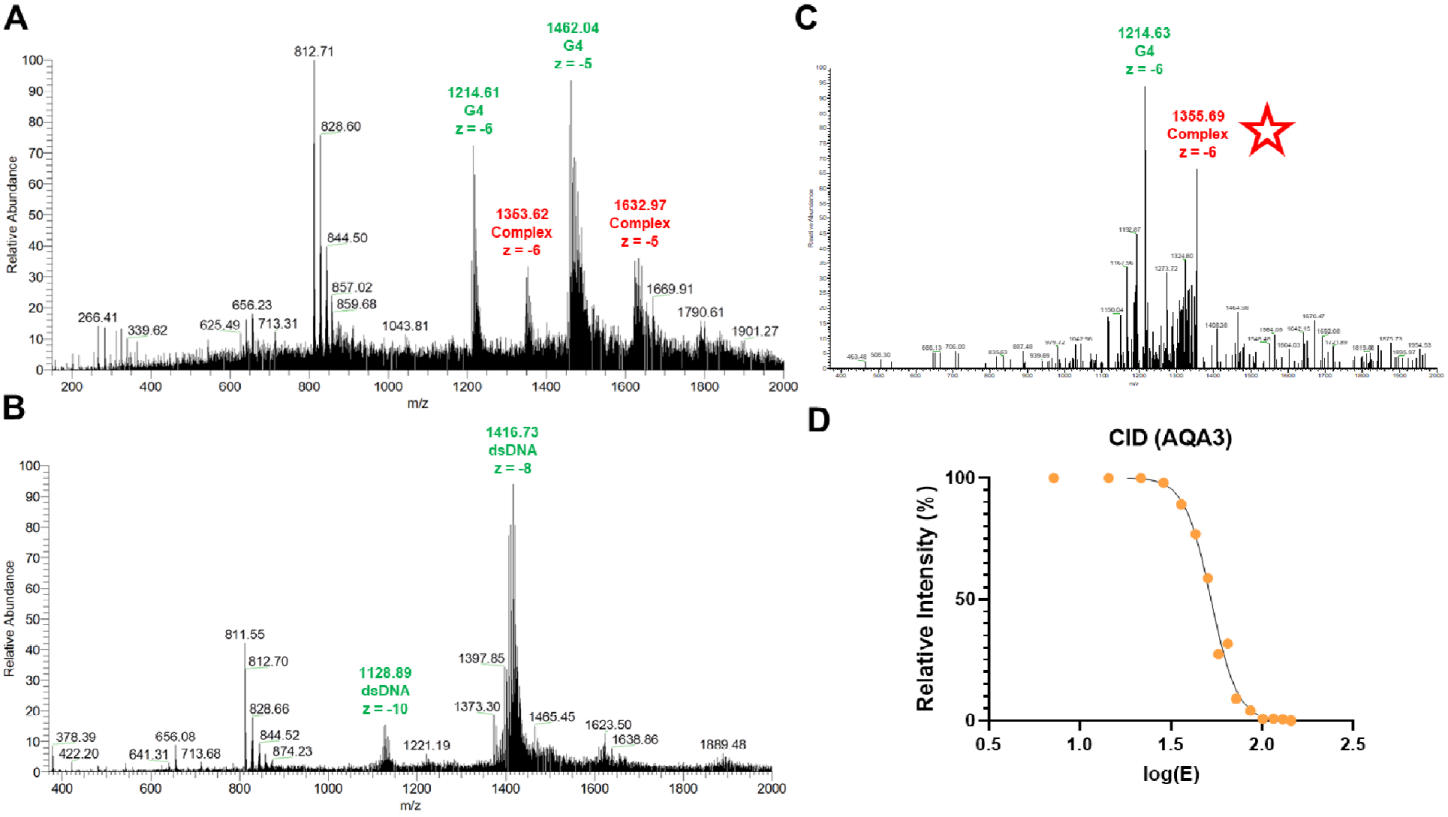
(A) ESI‐MS experiment showing the interaction of AQA3 with G‐quadruplex DNA Tel23 (G4). Peaks labeled in green represent G4 ions at different charge states (z), while the red ones represent Tel23‐AQA3 complex ions (z = ‐5 and z = ‐6; “Complex”); (B) ESI‐MS experiment investigating the interaction of AQA3 with dsDNA. Peaks labeled in green represent different charge states (z) of dsDNA. In this spectrum, it is possible to appreciate the absence of significant peaks showing the interaction between AQA3 and the DNA (z = ‐8 and z = ‐10); (C) CID experiment in which the dissociation of the Tel23‐AQA3 complex (red star) was promoted, leading to the appearance of the G4 peak (green); (D) Dissociation curve obtained by recording different spectra at increasing collision energy values.

In this work, ESI‐MS was performed in negative ionization mode using 150 mM ammonium acetate as buffer, and methanol was added to obtain a stable ESI signal [17, 26]. The compounds were tested toward the same intramolecular telomeric G4 (Tel23) and a double‐stranded DNA (dsDNA) sequence (Table 1 and Figure 2). MTX was used as a reference compound and the experimental output confirmed its preferential interaction with dsDNA (BA G4/BA dsDNA ratio = 0.60), thus validating the method [29].

**TABLE 1:** Binding affinity values (BA) of the tested compounds toward G4 and dsDNA and their selectivity ratio. n.c. = not calculable. BA values were not calculated for interaction peaks showing relative signal intensities lower than 5% in the mass spectrum. As a consequence, also the selectivity ratio is expressed as n.c. = not calculable.

	BA G4	BA dsDNA	G4 selectivity ratio [BA G4/BA dsDNA]
AQAp	–	–	–
AQA3m	–	–	–
AQA3	30	–	n.c.
ANAp	26	9	2.9
ANA3	–	–	–
Mitoxantrone	27	46	0.6

For the new derivatives, the ESI‐MS data reported in Table 1 indicated that among the tested anthraquinone derivatives, only AQA3 bound G4, while none of them interacted with dsDNA. Indeed, as reported in Figure 2A, B, AQA3 formed an adduct only with Tel23 in the MS spectrum. This indicated AQA3 as a selective binder for the telomeric G4. As it concerns the anthracene‐based derivatives, this approach identified ANAp as a weaker and poorly selective G4 binder, while it confirmed ANA3 as unable to interact with both tested DNA sequences.

The promising results obtained with AQA3 prompted us to further investigate its interaction with the nucleic acid by performing collision‐induced dissociation (CID) studies of AQA3 with Tel23 G4. CID is a mass spectrometry technique that allows an energy‐dependent fragmentation of selected ions in the gas phase. In our experiment, this ion is the DNA‐ligand complex that is exposed to growing normalized collision energy (NCE) values, promoting dissociation of the ligand (Figure 2C, D). The energy required for the dissociation of the complex peak to its half‐intensity is defined as E_COM_^50%^. For AQA3, an E_COM_^50%^ value of 52.4 eV was retrieved. This result demonstrated that this compound forms a stable interaction with the nucleic acid, which is even stronger than those previously measured for anthracene and anthraquinone derivatives by our group [17, 25].

**FIGURE 3:**
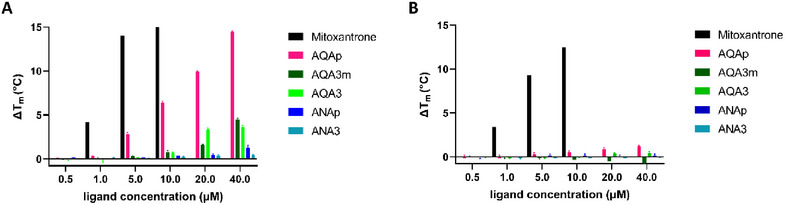
Fluorescence thermal shift experiments. Variation of the melting temperature induced by increasing concentrations of the analyzed monomeric (AQAp, ANAp) and dimeric (AQA3 and ANA3) derivatives on (A) Tel23‐FD and (B) scr‐dsDNA in 10 mM Li_3_(PO)_4_, 50 mM KCl, pH 7.5.

Finally, to fully compare the behavior of these new derivatives with the first set of monomeric building blocks, they were further analyzed by FTSA (Figure 3). Unexpectedly, the obtained results were not fully in line with the mass spectrometry output. While both experimental approaches confirmed that no compounds of this dataset stabilized dsDNA, under FTSA experimental conditions, only anthraquinone‐based derivatives stabilized the telomeric G4. Additionally, AQAm and AQA3 resulted to be less effective with reference to AQAp.

### 2.3 Characterization of AQAp and AQA3 Interaction with G4

FTSA needs to be performed under conditions that do not maximize the stability of G4 in the absence of ligands. As a result, the observed thermal shifts are not related only to the BA of the tested ligand to the target, but they are also a function of the DNA topological rearrangements induced by the complex formation. To address whether a balance of these mechanisms might play a role in explaining the divergent results described above, we performed Circular Dichroism (CD) titrations to compare the interaction of AQAp and AQA3 with Tel23 under conditions that promoted its folding into a stable G4 arrangement.

**FIGURE 4:**
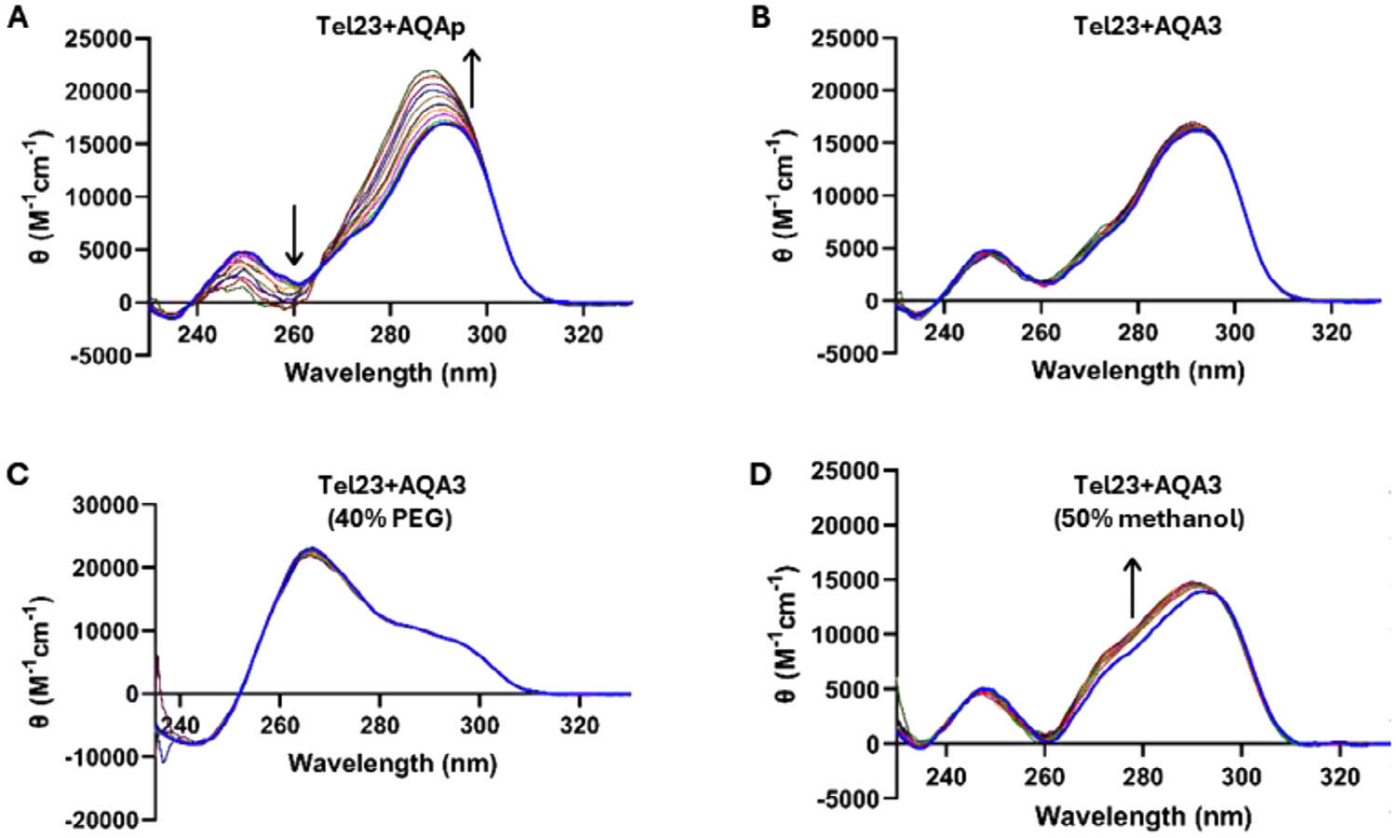
CD Titrations. Variation of the CD spectrum of Tel23 recorded in (A) 5 mM TRIS, 150 mM KCl, pH 7.5 upon addition of AQAp; (B) 5 mM TRIS, 150 mM KCl, pH 7.5 upon addition of AQA3; (C) 5 mM TRIS, 150 mM KCl, pH 7.5, 40% PEG‐200 upon addition of AQA3 and (D) 75 mM ammonium acetate, 50% MeOH upon addition of AQA3.

At first, we increased the KCl concentration up to 150 mM. As reported in Figure 4A,B, under this condition, the chiroptical profile of Tel23 fits with its folding into a Type 2‐hybrid G4 [4]. The addition of AQAp induced a significant increment of the positive contribution centered at 290 nm. By analyzing the variation of the CD signal at this wavelength as a function of AQAp concentration, we derived an EC_50_ = 37.7 ± 2.2 µM, thus confirming its good interaction with Tel23. Conversely, no chiroptical changes were observed upon addition of AQA3, further supporting its inability to bind G4. Overall, both results were in line with the FTSA data.

**FIGURE 5:**
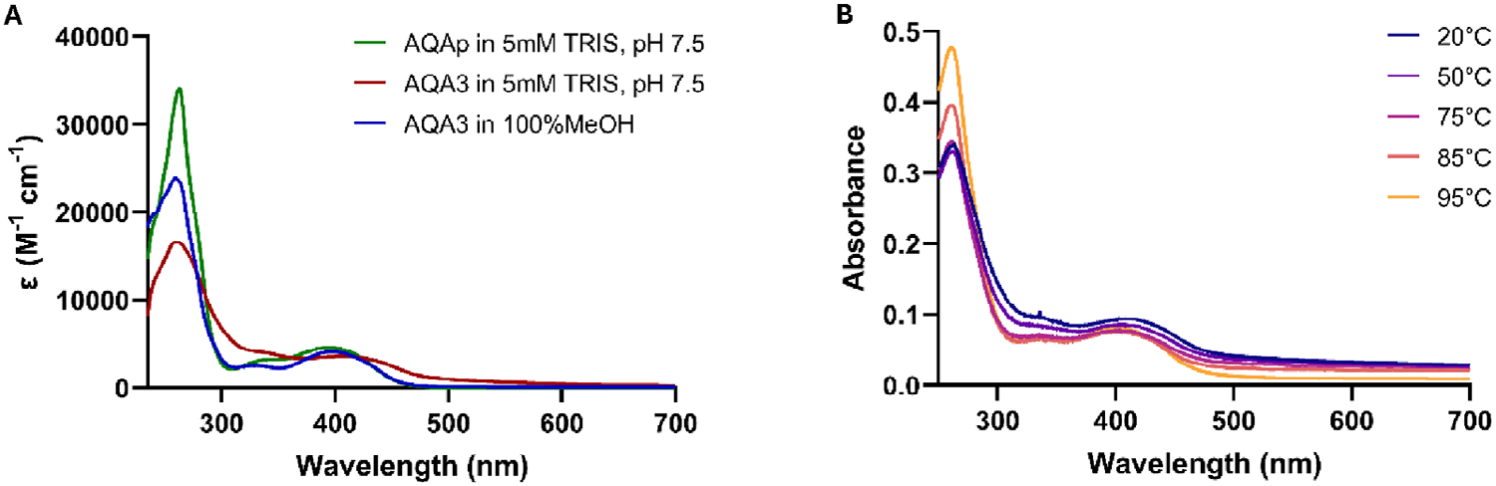
UV‐VIS absorption profiles of (A) 30 µM AQA3 acquired in methanol (blue line) or in 5 mM TRIS, pH 7.5 (red line), compared to 30 µM AQAp acquired in 5 mM TRIS, pH 7.5 (green line), and of (B) 30 µM AQA3 acquired in 5 mM TRIS, pH 7.5, at different temperatures.

Additionally, we took into account that the experimental conditions applied for FTSA and CD were remarkably different from those required in ESI‐MS. They can promote the folding of the target telomeric G4 into different topologies that tested ligands can recognize with modulated efficiency. To validate whether this might be the rationale for the different outputs derived among the different screening approaches, we used AQA3 to titrate Tel23 in 40% PEG‐200, where it folds into a parallel G4 which, from docking analyses should represent the most favorable topology for the tweezer binding mode (Figure 4C) [30], and in ammonium acetate with 50% MeOH, to mimic the experimental conditions applied for native MS analyses and where the hybrid arrangement is retained although with a small reduction of the antiparallel component (Figure 4D). Under both conditions, AQA3 did not induce significant variations in the chiroptical profile of Tel23, further pointing to its poor interaction with the G4 structure. Still, we paid attention to the modest changes observed in the presence of methanol, which we related to the odd spectroscopic behavior of AQA3. Indeed, the UV‐Vis absorption profile of AQA3 was different from the one of AQAp, although they share the same chromophoric unit (Figure 5). Conversely, when the UV‐Vis spectra of AQA3 were acquired at high temperature or in methanol, they started to match those of AQAp. Based on the hypochromic and bathochromic effect observed for the dimeric derivative in buffer, we inferred the occurrence of stacking interactions of the aromatic units for AQA3. Since this profile was conserved within the entire tested concentration range (0‐40 µM), while it never occurred for the monomeric AQAp, we can tentatively associate it to a fold‐back of the two aromatic units of AQA3 promoted by the flexible linker through a proximity effect, which may impair efficient G4 recognition in water solution.

### 2.4 Computational Studies

**FIGURE 6:**
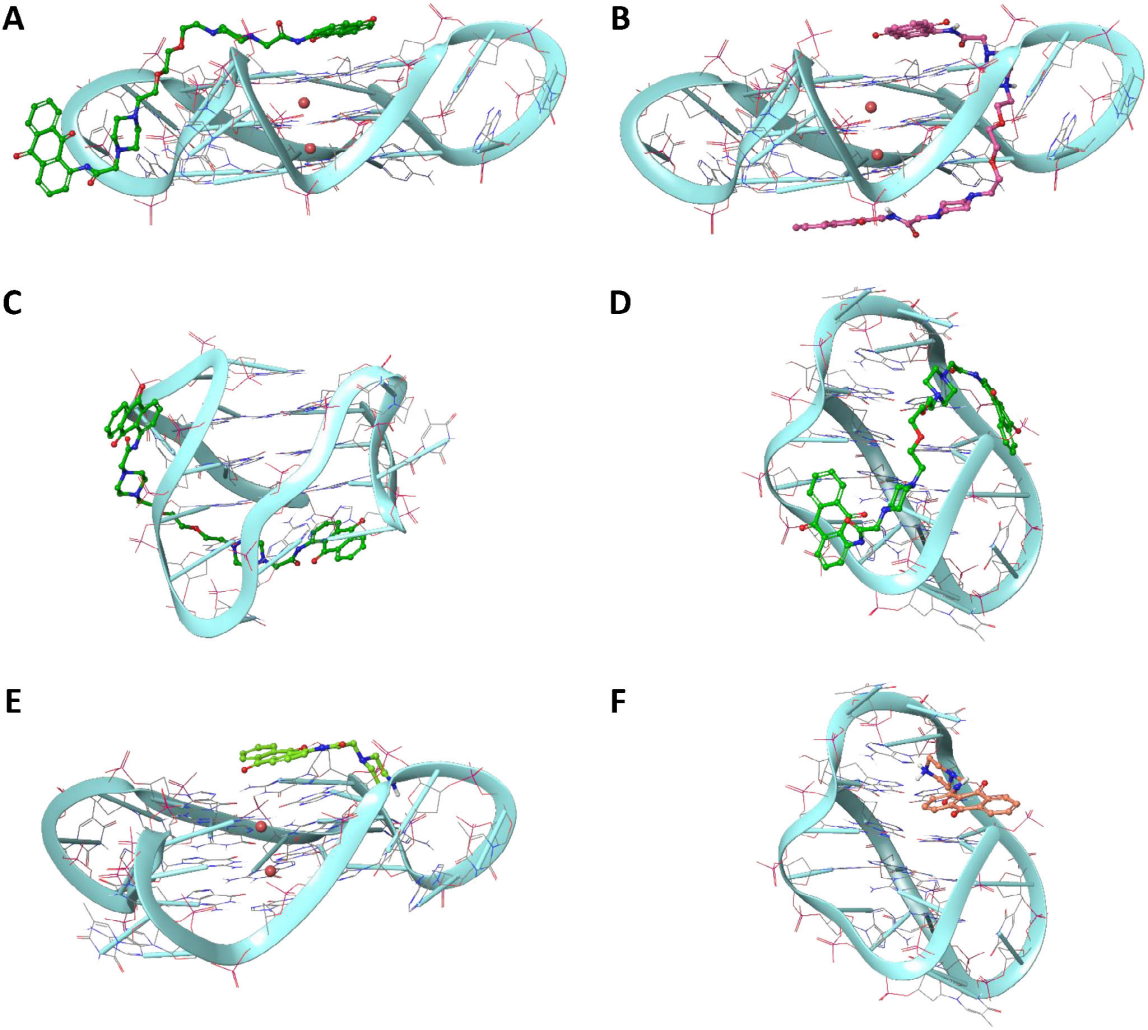
Overview of the docking poses of the complexes of AQA3 with: (A) 1KF1 in “hugger” binding mode (GlideScore = ‐9.609 kcal/mol); (B) 1KF1 in tweezer binding mode (GlideScore = ‐10.225 kcal/mol); (C) 2JPZ (GlideScore = ‐10.795 kcal/mol); and (D) 143D (GlideScore = ‐8.288 kcal/mol). Overview of the docking poses of the complexes of AQAp with: (E) 1KF1 in stacking binding mode (GlideScore = ‐6.959 kcal/mol); and (F) 143D in groove binding mode (GlideScore = ‐5.179 kcal/mol). No docking poses were generated for the 2JPZ topology.

To further provide insights for the interpretation of experimental results, molecular docking was employed to unravel putative binding modes of AQA3, which was addressed as a selective G4 binder by ESI‐MS, and of its monomeric counterpart AQAp, that was the most efficient in water solution. To comprehensively investigate the ligand‐G4 interaction at a molecular level, docking was conducted toward 1KF1 [31], 2JPZ, [32] and 143D [33] PDB structures, corresponding to a parallel, hybrid‐2 and antiparallel topology, respectively. The results of docking simulations are summarized in Figure 6.

Theoretically, AQA3 was designed to bind G4 structures with a molecular tweezer‐like interaction, binding the guanines that form G‐tetrads through aromatic stacking interactions, with piperazines interacting with the grooves. Theoretically, AQA3 was designed to bind G4 structures with a molecular tweezer‐like interaction, binding the guanines that form G‐tetrads through aromatic stacking interactions, with piperazines interacting with the grooves. In agreement, AQA3 behaved as predicted by the rationale design with the 1KF1 model (docking score computed for this pose: ‐10.286 kcal/mol). This was not the case for 2JPZ (‐10.795 kcal/mol) and 143D (‐8.288 kcal/mol), suggesting that a longer spacer may be required by the ligand to properly adopt the tweezer binding mode on these structures and highlighting a less efficient binding to 143D.

Furthermore, it must be pointed out that an additional binding mode for AQA3 in complex with 1KF1 was identified (defined as hugger‐like, AQA3h). The reader can find a representation of all obtained docking poses clustered in the main binding motifs in the Supporting Information. To compare this binding mode to the tweezer one (AQA3t) we mapped their interaction network with parallel G4. Specifically, within AQA3t‐1KF1 complex, each anthraquinone unit interacted through two π‐π stacking interactions with guanines (DG) of the two outer tetrads, namely DG8 (3.66 and 3.76 Å, respectively) and DG16 (3.79 and 4.21 Å, respectively). Other stabilizing interactions involved one piperazine unit, which formed one hydrogen bond (2.31 Å) and one salt bridge (3.03 Å) with DG21, located in the central tetrad. Additionally, another hydrogen bond was seen between G14 and the secondary amide in the linker (2.57 Å). In contrast, in AQA3h only one anthraquinone moiety formed π‐π stacking interactions with DG16 present on the external tetrad in 1KF1 (3.97, 4.28, and 4.39 Å, respectively). In this case, the piperazine moieties in AQA3h were able to establish, in one instance, one hydrogen bond and one salt bridge with DG9 (1.80 and 2.73 Å, respectively). In contrast, in another pose, there was one π‐cation interaction with DG4 (4.19 Å) (See Supplementary Information). Concerning AQAp, the anthraquinone moiety established three π‐π stacking interactions with DG16 (3.59 and 3.98 Å) and DG22 (3.79 Å) while, for the piperazine ring, one nitrogen interacted with DG21 via a salt bridge and a hydrogen bond (1.62 and 2.54 Å) and the other one was involved in an additional hydrogen bond with DG16 (2.65 Å).

**FIGURE 7:**
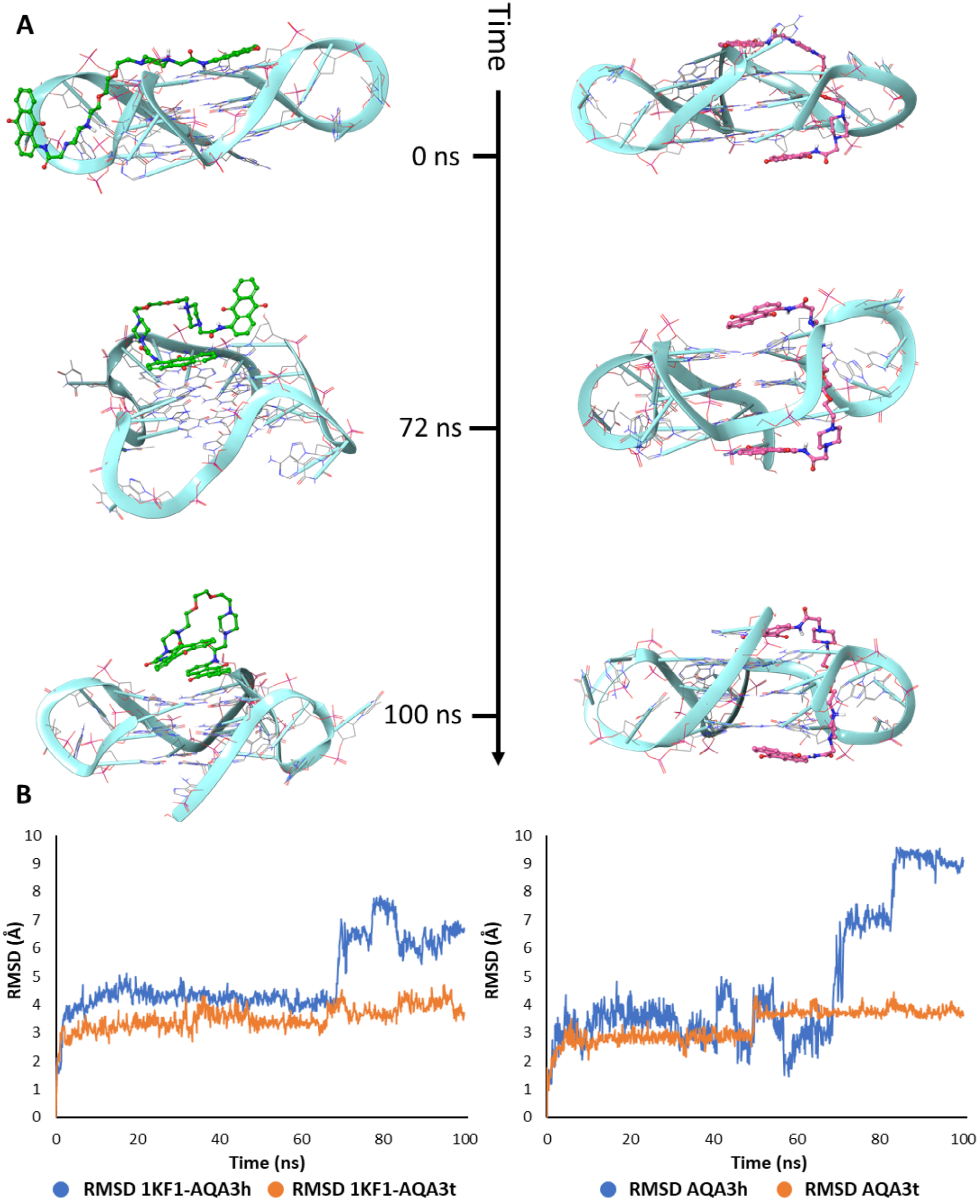
(A) Snapshots of the 1KF1‐AQA3h complex in the “hugger” binding mode (on the left) and of 1KF1‐AQA3t complex in the tweezer‐like binding mode (on the right) are presented relative to frame 1 (0 ns), frame 735 (72 ns), and frame 1000 (100 ns); (B) Depiction of trajectories of 1KF1‐AQA3h and 1KF1‐AQA3t over 100 ns of MD simulation, RMSD of 1KF1 bound to AQA3h (blue) and to AQA3t (orange), and RMSD of ligands for AQA3h and AQA3t.

To better clarify the stability of the different binding modes of AQA3, Molecular Dynamic (MD) simulations were performed using two starting points: 1KF1‐AQA3 in “hugger”‐like mode (1KF1‐AQA3h) and 1KF1‐AQA3 in tweezer‐like mode (1KF1‐AQA3t). Trajectories were analyzed using the RMSD parameter to gain insights into the stability of 1KF1, AQA3h, and AQA3t. This revealed that the telomeric parallel G4 (1KF1) was more stabilized by hydrogen bonds and π‐π interactions with AQA3 in a tweezer‐like binding mode, supporting the rationale of the work. On the contrary, the “hugger”‐like binding mode of AQA3 lost partial interactions with G4 after 72 ns of simulation, leading to a lower stability of the structure (Figure 7). By evaluating these results, it is possible to deduce that the proposed “tweezer‐like” binding mode is confirmed to be a good stabilizing strategy for the design of parallel G4s‐binders.

### 2.5 Evaluation of the Cytotoxic Activity of Anthraquinone‐Based Derivatives

To preliminarily address whether the so far described different behavior of AQAp and AQA3 in solution might drive variable cellular effects, we evaluated their cytotoxicity on a human gastric carcinoma cell line (HGC‐27). To properly observe both short and long‐term effects, ligand treatments were performed for 24 and 72 h (see Supporting Information). The obtained IC_50_ values are reported in Table 2.

**TABLE 2:** IC_50_ values calculated on the HGC‐27 cells treated for 24 and 72 h with increasing concentrations of AQAp and AQA3.

	24 h	72 h
AQAp	9.9 ± 0.6 µM	3.3 ± 0.1 µM
AQA3	23.1 ± 1.6 µM	7.2 ± 0.6 µM

At both treatment times, AQAp exhibited IC_50_ values almost twofold lower compared to AQA3 in the tested HCG‐27 cell line. It might be tempting to relate this difference to the above‐reported preferential G4 binding of AQAp vs AQA3 above determined in water solution. Whether this holds true, it would suggest that also in the cellular environment, the dimeric compound is not fully available to interact with G4s. However, it must be highlighted that the chemical‐physical properties of this compound can impact several physiologically relevant steps beside G4 recognition. Indeed, we showed that the self‐stacked form of AQA3 is highly stable and, moreover, it was predicted to be more lipophilic than the monomeric derivative (LogP 2.87 and 0.81 for AQA3 and AQAp, respectively). Thus, we cannot rule out that these features can modulate AQA3 cellular uptake and/or intracellular distribution, ultimately resulting in a reduced effective drug concentration at functional target sites, including G4. On the other hand, the results obtained with AQAp confirmed the potential application of anthraquinone‐based ligands as G4 targeting agents.

## 3 Experimental Section

### 3.1 Chemicals and Instruments

Commercially available chemicals were purchased from Merck or VWR and used without any further purification if not specified elsewhere. NMR experiments were performed on a Bruker (Bruker, Billerica, MA) Ascend spectrometer (frequencies: 400.13 and 100.62 MHz for ^1^H and ^13^C nuclei, respectively) equipped with a multinuclear inverse z‐field gradient probe head (5 mm). For data processing, TopSpin 4.4.1 software was used, and the spectra were calibrated using solvent signal (^1^H‐NMR, δ_H_ = 7.26 ppm for CDCl_3_, δ_H_ = 3.31 ppm for CD_3_OD, δ_H_ = 2.50 ppm for DMSO‐*d6*; ^13^C‐NMR, δ_C_ = 77.16 ppm for CDCl_3_, 39.52 ppm for DMSO‐*d6*, δ_C_ = 49.00 ppm for CD_3_OD). Multiplicities are reported as follows: s, singlet; d, doublet; t, triplet; q, quartet; m, multiplet; b, broad; dd, doublet of doublets. Mass spectra were recorded by direct infusion ESI on a LCQ Fleet ion trap (Thermo Fisher Scientific, Waltham, MA, USA) mass spectrometer. For data processing, Qual Browser Thermo Xcalibur 4.0.27.13 software was used. ESI parameters for samples acquired in positive ionization mode: spray voltage 3.2 kV, capillary temperature 250°C, capillary voltage 43 V. The purity profile was assayed by HPLC using a Pro‐Star system (Palo Alto, CA, USA) equipped with a 1706 UV‐VIS detector (Bio‐Rad, Hercules, CA, USA) and a C‐18 column (5 µm, 4.6 × 150 mm) (Agilent, Santa Clara, CA, USA). An appropriate gradient of 0.1% formic acid (A) and acetonitrile (B) was used as mobile phase with an overall flow rate of 1 mL min^−1^. The general methods for the analyses are reported in the following: 0 min (90% A−10% B), 2 min (90% A−10% B), 10 min (5% A−95% B), 14 min (5% A−95% B), and 16 min (90% A−10% B). Analyses were performed at 254 nm and purity profile was above 95% for final compounds (area %).

### 3.2 Synthesis of AQA (2‐bromo‐N‐(9, 10‐dioxo‐9, 10‐dihydroanthracen‐1‐yl)acetamide)

A one‐neck round‐bottom flask was loaded with 1‐aminoanthraquinone (100 mg, 0.45 mmol) and K_2_CO_3_ (62 mg, 0.45 mmol) in 3 mL of acetonitrile (AcCN). Bromoacetyl bromide (51 µL, 0.585 mmol) was then added dropwise to the reaction mixture. The reaction was monitored with thin layer chromatography (TLC) with the proper eluent (hexane:ethyl acetate, 2:1) and HPLC. After 5 h, bromoacetyl bromide (25.5 µL, 0.29 mmol) was added and the reaction was stirred overnight. After checking the progress with TLC, the following day another aliquot of bromoacetyl bromide (25.5 µL, 0.29 mmol) was added, and the reaction was then quenched by adding 10 mL of H_2_O. The solid that precipitated was filtered with a Büchner funnel and then washed with ethanol (EtOH) (5 mL) and dried with hexane (3*2 mL) in order to remove the excess of starting materials and possible byproducts. The solid was then retrieved from filter with a spatula and collected in an Eppendorf tube. Yield: 49%. ^1^H‐NMR (400.13 MHz, CDCl_3_) δ_H_ (ppm): 12.94 (s, 1H, NH), 9.14 (dd, J_1_ = 1.1 Hz, J_2_ = 8.5 Hz, 1H, Ar‐H), 8.35‐8.28 (m, 2H, Ar‐H), 8.08 (dd, J_1_ = 1.13 Hz, J_2_ = 7.63 Hz, 1H, Ar‐H), 7.83‐ 7.79 (m, 3H, Ar‐H), 4.10 (s, 2H, CH_2_). ESI‐MS m/z calculated for [C_16_H_11_BrNO_3_]^+^: 343.99, found: 343.15.

### 3.3 Synthesis of AQAp (N‐(9, 10‐dioxo‐9, 10‐dihydroanthracen‐1‐yl)‐2‐(piperazin‐1‐yl)acetamide)

Piperazine (253 mg, 2.9 mmol) and N, N‐dimethylformamide (DMF) (2 mL) were introduced in a round‐bottom flask, and the reaction mix was stirred and heated up to about 50°C in order to partially dissolve piperazine. After this step, the heating was turned off and AQA (100 mg, 0.29 mmol) was added. The reaction was monitored with TLC with the proper eluent (dichloromethane (DCM): methanol (MeOH): triethylamine (TEA), 92:7.5:0.5) and HPLC. The reaction was stirred for 4 h at room temperature, and then 10 mL of H_2_O were added to quench the reaction, the precipitate was filtered with a Büchner funnel in order to remove the excess of starting materials and possible byproducts. The solid was then retrieved from the filter with a spatula and collected in an Eppendorf tube. Yield: 78%. ^1^H‐NMR (400.13 MHz, CDCl_3_): δ_H_ (ppm): 13.21 (s, 1H, NH); 9.23 (d, 1H, J = 7.9 Hz, Ar‐H); 8.31 (dd, 2H, J_1_ = 6.5 Hz, J_2_ = 28.7, Ar‐H); 8.09 (d, 1H, J = 7.9 Hz, Ar‐H); 7.81‐7.75 (m, 3H, Ar‐H); 3.25 (s, 2H, CH_2_); 3.18 (b, 4H, 2*CH_2_); 2.66 (b, 4H, 2*CH_2_). ^13^C‐NMR (100.62 MHz, CDCl_3_): δ_C_ (ppm): 186.3 (C = O); 183.1 (C = O); 171.6 (C = O); 141.3 (Csp^2^); 135.6 (Csp^2^); 134.4 (Csp^2^); 134.3 (Csp^2^); 134.3 (Csp^2^); 132.9 (Csp^2^); 127.6 (Csp^2^); 127.1 (Csp^2^); 126.4 (Csp^2^); 122.8 (Csp^2^); 118.7 (Csp^2^); 63.6 (Csp^3^); 55.2 (Csp^3^); 46.0 (Csp^3^). ESI‐MS m/z calculated for [C_20_H_20_N_3_O_3_]^+^: 350.40, found 350.41.

### 3.4 Synthesis of AQApBut (2‐(4‐butylpiperazin‐1‐yl)‐N‐(9, 10‐dioxo‐9, 10‐dihydroanthracen‐1‐yl)acetamide)

1‐Chlorobutane (5.4 mg, 0.058 mmol), Cs_2_CO_3_ (18.9 mg, 0.058 mmol) and KI (9.6 mg, 0.058 mmol) were introduced in a reaction flask and then dissolved with 0.25 mL of DMF. The reaction mix was stirred for 15 min at room temperature to promote a Finkelstein reaction, which consists in the treatment of a primary alkyl halide (1‐chlorobutane) with an alkali metal halide (KI) leading to the replacement of the halogen via SN_2_ nucleophilic substitution. N‐(9, 10‐dioxo‐9, 10‐dihydroanthracen‐1‐yl)‐2‐(piperazin‐1‐yl)acetamide (AQAp; 8 mg, 0.023 mmol), was then added to the reaction mix to make it react with the newly formed butyl halide to form AQApBut. The reaction mix was stirred at room temperature and monitored with HPLC. After a week, the reaction was quenched with 2 mL of H_2_O and then centrifuged (10,000 rpm, 5 min). After the water removal, a second wash was performed with a 10% H_2_O /EtOH solution, and then the centrifugation procedure previously seen was repeated. n‐Hexane was then added to extract AQApBut from the solid. This separation method was repeated other two times. The three aliquots were then merged together, and the residual solvents were removed through the use of a vacuum concentrator. Yield: 35%. ^1^H‐NMR (400.13 MHz, CDCl_3_): δ_H_ (ppm): 13.16 (s, 1H, NH); 9.23 (dd, 1H, J_1_ = 1.0 Hz, J_2_ = 8.5 Hz, Ar‐H); 8.33‐8.26 (m, 2H, Ar‐H); 8.08 (dd, 1H, J_1_ = 1.1 Hz, J_2_ = 7.6 Hz Ar‐H); 7.83‐7.76 (m, 3H, Ar‐H); 3.27 (s, 2H, NCH_2_); 2.75 (b, 8H, 4*CH_2_); 2.48 (t, 2H, J = 15.5 Hz, NCH_2_); 1.59‐1.51 (m, 2H, CH_2_); 1.41‐1.35 (m, 2H, CH_2_); 0.96 (t, 3H, J = 14.8 Hz, CH_3_). ^13^C‐NMR (100.62 MHz, CDCl_3_): δ_C_ (ppm): 186.0 (C = O); 183.0 (C = O); 171.5 (C = O); 141.2 (Csp^2^); 135.4 (Csp^2^); 134.2 (Csp^2^); 134.2 (Csp^2^); 134.1 (Csp^2^); 134.1 (Csp^2^); 132.8 (Csp^2^); 127.3 (Csp^2^); 127.0 (Csp^2^); 126.4 (Csp^2^); 122.6 (Csp^2^); 118.5 (Csp^2^); 62.9 (Csp^3^); 59.5 (Csp^3^); 58.6 (Csp^3^); 53.7 (Csp^3^); 53.0 (Csp^3^); 31.6 (Csp^3^); 29.2 (Csp^3^); 22.7 (Csp^3^); 20.9 (Csp^3^); 14.2 (Csp^3^). A selective gradient TOCSY experiment (seldigpzs in Bruker library) was performed to assign the butyl peaks of AQApBut. The selective pulse was targeted at the butyl N‐CH2 peak signal and allowed the identification of the other butyl signals, since they are part of the same spin system. ESI‐MS m/z calculated for [C_24_H_28_N_3_O_3_]^+^: 406.21, found 406.28.

### 3.5 Synthesis of AQAep (N‐(9, 10‐dioxo‐9, 10‐dihydroanthracen‐1‐yl)‐2‐(4‐(2‐hydroxyethyl)piperazin‐1‐yl)acetamide)

2‐bromo‐N‐(9, 10‐dioxo‐9, 10‐dihydroanthracen‐1yl)acetamide (AQA; 20 mg, 0.06 mmol) was introduced in a reaction flask and then dissolved with 0.5 mL of N, N‐dimethylformamide (DMF). 1‐(2‐Hydroxyethyl)piperazine (123 µL, 0.24 mmol) was added and the reaction mix was stirred overnight at room temperature and monitored with HPLC. After 24 h, the reaction was quenched with 4 mL of H_2_O and then centrifuged (10,000 rpm, 5 min). After the water removal a second wash was performed with a 10% water/EtOH solution, and then the centrifugation procedure previously seen was repeated. EtOH was added to the tubes, and the residual solvents were removed through the use of a vacuum concentrator. Yield: 88%. ^1^H‐NMR (400.13 MHz, CDCl_3_): δ_H_ (ppm): 13.16 (s, 1H, NH); 9.22 (dd, 1H, J_1_ = 1.2 Hz, J_2_ = 8.5 Hz, Ar‐H); 8.32‐8.26 (m, 2H, Ar‐H); 8.08 (dd, 1H, J_1_ = 1.3 Hz, J_2_ = 7.8 Hz, Ar‐H); 7.84‐7.75 (m, 3H, Ar‐H); 3.68 (t, 2H, J = 10.6 Hz, CH_2_); 3.28 (s, 2H, NCH_2_); 2.83‐2.81 (m, 4H, 2*CH_2_); 2.75‐2.74 (m, 4H, 2*CH_2_); 2.70 (t, 2H, J = 10.6 Hz, NCH_2_). ^13^C‐NMR (100.62 MHz, CDCl_3_): δ_C_ (ppm): 186.2 (C = O); 183.0 (C = O); 171.5 (C = O); 141.3 (Csp^2^); 135.6 (Csp^2^); 134.4 (Csp^2^); 134.3 (Csp^2^); 134.2 (Csp^2^); 132.9 (Csp^2^); 127.4 (Csp^2^); 127.1 (Csp^2^); 126.5 (Csp^2^); 122.8 (Csp^2^); 118.7 (Csp^2^); 62.9 (Csp^3^); 59.5 (Csp^3^); 58.0 (Csp^3^); 53.8 (Csp^3^); 52.8 (Csp^3^). ESI‐MS m/z calculated for [C_22_H_24_N_3_O_4_]^+^: 394.18, found 394.29.

### 3.6 Synthesis of AQAbp (tert‐butyl 4‐(2‐((9, 10‐dioxo‐9, 10‐dihydroanthracen‐1‐yl)amino)‐2‐oxoethyl)piperazine‐1‐carboxylate)

2‐bromo‐N‐(9, 10‐dioxo‐9, 10‐dihydroanthracen‐1yl)acetamide (AQA; 128 mg, 0.37 mmol) was introduced in a reaction flask and then dissolved with 8 mL of DMF. TEA (187 µL, 1.34 mmol) and 1‐Boc‐piperazine (208 mg, 1.12 mmol) were added, and the reaction mix was left to stir at room temperature and monitored with HPLC. After 4 h, the reaction was quenched with cold H_2_O and then filtered with a Büchner funnel, obtaining a yellowish solid. The solid was then retrieved from the filter with a spatula and collected in an Eppendorf tube. The residual solvents were removed through the use of a vacuum concentrator. Yield: 48%. ^1^H‐NMR (400.13 MHz, CDCl_3_): δ_H_ (ppm): 13.23 (s, 1H, NH); 9.22 (dd, 1H, J_1_ = 1.3 Hz, J_2_ = 8.5 Hz, Ar‐H); 8.35‐8.27 (m, 2H, Ar‐H); 8.10 (dd, 1H, J_1_ = 1.2 Hz, J_2_ = 7.6 Hz, Ar‐H); 7.85‐7.76 (m, 3H, Ar‐H); 3.74 (b, 4H, 2*CH_2_); 1.93 (s, 2H, CH_2_); 2.66 (b, 4H, 2*CH_2_); 1.50 (s, 9H, 3*CH_3_). ^13^C‐NMR (100.62 MHz, CDCl_3_): δ_C_ (ppm): 186.5 (C = O); 183.0 (C = O); 154.9 (Csp^2^); 141.2 (Csp^2^); 135.6 (Csp^2^); 134.4 (Csp^2^); 134.4 (Csp^2^); 134.3 (Csp^2^); 134.2 (Csp^2^); 132.9 (Csp^2^); 127.6 (Csp^2^); 127.1 (Csp^2^); 126.4 (Csp^2^); 122.9 (Csp^2^); 118.7 (Csp^2^); 80.0 (Csp^3^); 63.0 (Csp^3^); 53.6 (Csp^3^); 28.6 (Csp^3^). ESI‐MS m/z calculated for [C_25_H_28_N_3_O_5_]^+^: 450.20, found 450.24.

### 3.7 Synthesis of AQA3 (2, 2'‐(((ethane‐1, 2‐diylbis(oxy))bis(ethane‐2, 1‐diyl))bis(piperazine‐4, 1‐diyl))bis(N‐(9, 10‐dioxo‐9, 10‐dihydroanthracen‐1‐yl)acetamide)) and of its AQAp‐based mono‐substituted analogue AQA3m (N‐(9, 10‐dioxo‐9, 10‐dihydroanthracen‐1‐yl)‐2‐(4‐(2‐(2‐(2‐hydroxyethoxy)ethoxy)ethyl)piperazin‐1‐yl)acetamide)

A one‐neck round‐bottom flask was charged with 1 mL of DMF and Cs_2_CO_3_ (145 mg, 0.45 mmol) and then heated up to about 50°C in order to partially dissolve the cesium carbonate. The heating was then turned off and AQAp (65 mg, 0.19 mmol) and 1, 2‐bis(2‐iodoethoxy)ethane (PEG3‐I) (13.6 µL, 0.07 mmol) were added to the mixture and it was stirred overnight. The following day were added 0.2 equivalents of PEG3‐I (6.8 µL, 0.04 mmol). The reaction was monitored with TLC with the proper eluent (DCM:MeOH:TEA, 92:7.5:0.5) and HPLC. The reaction was then stopped after 4 h, and 10 mL of H_2_O were added to quench the reaction. Subsequently, the precipitate was transferred into a falcon tube and then centrifuged at 4000 g for 5 min. This step was repeated 3 times. Afterwards, a chromatographic column was performed. AQA3 was charged with the dry loading method, by adsorbing the compound on silica powder, and after tests with different eluents it was chosen DCM:MeOH (9:1). After the column were isolated 4 fractions, and the compound was further purified with two preparative TLC with DCM:MeOH (94:6) as eluent. Four bands were collected by scraping the silica off the TLC sheets and the isolated compounds were extracted with 1 mL of DCM:MeOH (1:1); a centrifugation (10,000 rpm, 5 min) was performed to separate the organic phase from the silica and the four aliquots were then characterized with ESI‐MS, and the first band was identified as AQA3. The fractions of interest were then characterized with HPLC, ESI‐MS and ^1^H and ^13^C NMR spectroscopy. AQA3: yield: 16%. ^1^H‐NMR (400.62 MHz, CDCl3): δ_H_ (ppm): 13.19 (s, 1H, NH); 13.14 (s, 1H, NH); 9.21‐9.17 (m, 2H, Ar‐H); 8.30‐8.23 (m, 4H, Ar‐H); 8.08‐8.04 (m, 2H, Ar‐H); 7.81‐7.73 (m, 6H, Ar‐H); 3.82‐3.79 (m, 4H, 2*CH_2_); 3.76‐3.64 (m, 10H, 5*CH_2_); 3.26 (s, 2H, CH_2_); 3.25 (s, 2H, CH_2_); 2.83 (m, 4H, 2*CH_2_); 2.78‐2.75 (m, 6H, 3*CH_2_); 2.68‐2.66 (m, 4H, 2*CH_2_). ^13^C‐NMR (100.62 MHz, CDCl_3_): δ_C_ (ppm): 183.0 (C = O); 171.5 (C = O); 170.9 (C = O); 155.5 (Csp^2^); 141.3 (Csp^2^); 135.7 (Csp^2^); 135.6 (Csp^2^); 134.4 (Csp^2^); 134.3 (Csp^2^); 132.8 (Csp^2^); 127.5 (Csp^2^); 127.4 (Csp^2^); 127.1 (Csp^2^); 126.4 (Csp^2^); 126.4 (Csp^2^);122.9 (Csp^2^); 122.8 (Csp^2^); 118.6 (Csp^2^); 70.7 (Csp^3^); 70.6 (Csp^3^); 69.8 (Csp^3^); 64.7 (Csp^3^); 62.9 (Csp^3^); 58.0 (Csp^3^); 53.6 (Csp^3^); 53.5 (Csp^3^); 29.8 (Csp^3^). ESI‐MS: calculated for [C_46_H_48_N_6_Na_2_O_8_]^+^ 858.33, found 857.93. AQA3m: yield: 6%. ^1^H‐NMR (400.62 MHz, CDCl_3_): δ_H_ (ppm): 13.17 (s, 1H, NH); 9.23 (dd, 1H, J_1_ = 1.0 Hz, J_2_ = 8.7 Hz, Ar‐H); 832‐8.28 (m, 2H, Ar‐H); 8.08 (s, 1H, Ar‐H); 7.83‐7.78 (m, 3H, Ar‐H); 3.76‐3.60 (m, 12H, 6*CH_2_); 3.29 (s, 2H, CH_2_); 2.97‐2.82 (m, 8H, 4*CH_2_). ^13^C‐NMR (100.62 MHz, CDCl_3_): δ_C_ (ppm): 186.4 (C = O); 183.0 (C = O); 171.3 (C = O); 141.3 (Csp^2^); 135.6 (Csp^2^); 134.4 (Csp^2^); 134.3 (Csp^2^); 134.3 (Csp^2^); 132.9 (Csp^2^); 129.9 (Csp^2^); 127.4 (Csp^2^); 127.1 (Csp^2^); 126.5 (Csp^2^); 122.8 (Csp^2^); 118.6 (Csp^2^); 72.8 (Csp^3^); 70.5 (Csp^3^); 70.4 (Csp^3^); 62.7 (Csp^3^); 61.8 (Csp^3^); 29.8 (Csp^3^). ESI‐MS m/z calculated for [C_26_H_32_N_3_O_6_]^+^: 482.23, found 482.76.

### 3.8 Synthesis of ANA (N‐(anthracen‐2‐yl)‐2‐Bromoacetamide)

A one‐neck round‐bottom flask was charged with 2‐aminoanthracene (100 mg, 0.52 mmol) and 3.5 mL of AcCN. K_2_CO_3_ (72 mg, 0.52 mmol) and bromoacetyl bromide (58.9 µL, 0.68 mmol) were then added dropwise to the flask and the reaction mixture was stirred at room temperature overnight. The reaction was monitored with TLC with the proper eluent (hexane:ethyl acetate, 2:1) and HPLC. The following day, the starting material appeared consumed, and 10 mL of H_2_O were added in order to quench the reaction, the precipitate was filtered with a Büchner funnel and then washed with ethanol (5 mL) and dried with hexane (3*2 mL) in order to remove the excess of starting materials and possible byproducts. The solid was then retrieved from the filter with a spatula and collected in an Eppendorf tube. Yield: 74%. ^1^H‐NMR (400.13 MHz, DMSO‐*d6*) δ_H_ 10.58 (s, 1H, NH), 8.46‐8.43 (m, 3H, Ar‐H), 8.02‐7.98 (m, 3H, Ar‐H), 7.50‐7.48 (m, 1H, Ar‐H), 7.44‐7.41 (m, 2H, Ar‐H), 4.07 (s, 2H, CH_2_); ESI‐MS *m/z* calculated for [C_16_H_13_BrNO]^+^: 314.02, found: 314.34.

### 3.9 Synthesis of ANAp (N‐(anthracen‐2‐yl)‐2‐(piperazin‐1‐yl)acetamide)

Piperazine (332 mg, 3.85 mmol) and DMF (2 mL) were introduced in a round‐bottom flask, and the reaction mix was stirred and heated up to about 50°C in order to partially dissolve piperazine. After this step the heating was turned off and ANA (121 mg, 0.39 mmol) was added, and the reaction was stirred for 10 min. The reaction was monitored with TLC with the proper eluent (DCM:MeOH:TEA, 92:7.5:0.5) and HPLC. The reaction was stopped and 15 mL of ethyl acetate were added, the mixture was washed with NaOH 1 M (3*10 mL) in a separatory funnel and the organic phase was collected. The solution was then dried over anhydrous sodium sulfate and furtherly evaporated under reduced pressure. Yield: 99%. ^1^H‐NMR (400.13 MHz, CDCl_3_): δ_H_ (ppm): 8.84 (s, 1H, NH); 8.43 (s, 1H, Ar‐H); 8.38 (s, 2H, Ar‐H), 8.01‐7.97 (m, 3H, Ar‐H); 7.49‐7.43 (m, 3H, Ar‐H); 3.36‐3.34 (m, 6H, 3*CH_2_); 2.98‐2.96 (m, 4H, CH2). ^13^C‐NMR (100.62 MHz, CDCl_3_): δ_C_ (ppm): 168.8 (C = O); 134.4 (Csp^2^); 132.4 (Csp^2^); 132.1 (Csp^2^); 131.3 (Csp^2^); 129.5 (Csp^2^); 129.4 (Csp^2^); 128.3 (Csp^2^); 128.1 (Csp^2^); 126.3 (Csp^2^); 125.8 (Csp^2^); 125.8 (Csp^2^); 125.2 (Csp^2^); 120.5 (Csp^2^); 115.3 (Csp^2^); 62.9 (Csp^3^); 54.9 (Csp3); 46.5 (Csp^3^); 29.8 (Csp^3^). ESI‐MS: calculated for [C_20_H_22_N_3_O]^+^ 320.18, found 320.25.

### 3.10 Synthesis of ANA3 (2, 2'‐(((ethane‐1, 2‐diylbis(oxy))bis(ethane‐2, 1‐diyl))bis(piperazine‐4, 1‐diyl))bis(N‐(anthracen‐2‐yl)acetamide))

This reaction was performed in a one‐neck round‐bottom flask that was charged with 1 mL of DMF, Cs_2_CO_3_ (94 mg, 0.29 mmol), ANAp (60 mg, 0.19 mmol) and PEG3‐I (14.8 µL, 0.08 mmol) and stirred at 100°C. The reaction was monitored with TLC with the proper eluent (DCM:MeOH:TEA, 92:7.5:0.5) and HPLC. The reaction was stopped after 2 h, and 10 mL of H_2_O were added to quench the reaction. Subsequently, the precipitate was transferred into a falcon tube and then centrifuged at 4000 g for 5 min. This step was repeated 3 times. ANA3 was then washed with a solution of MeOH:H_2_O (3:7) and centrifuged with the same parameters applied before (3 times). The solid fraction was collected after removal of the liquid. The compound was obtained by performing a preparative TLC with a proper eluent (DCM:MeOH:TEA, 92:7.5:0.5). The product was then characterized with HPLC, ESI‐MS and ^1^H and ^13^C NMR. Yield: 11%. ^1^H‐NMR (400.13 MHz, CDCl_3_): δ_H_ (ppm): 9.31 (s, 2H, NH); 8.45 (s, 2H, Ar‐H); 8.36 (d, 4H, J = 8.4 Hz, Ar‐H); 7.98‐7.95 (m, 6H, Ar‐H); 7.46‐7.40 (m, 6H, Ar‐H); 3.67‐3.64 (m, 8H, 4*CH_2_); 3.20 (s, 4H, 2*CH_2_); 2.71‐2.61 (m, 20H, 10*CH_2_). ^13^C‐NMR (100.62 MHz, CDCl_3_): δ_C_ (ppm): 168.7 (C = O); 134.4 (Csp^2^); 132.4 (Csp^2^); 132.1 (Csp^2^); 131.3 (Csp^2^); 129.4 (Csp^2^); 129.4 (Csp^2^); 128.3 (Csp^2^); 128.1 (Csp^2^); 126.3 (Csp^2^); 125.8 (Csp^2^); 125.8 (Csp^2^); 125.2 (Csp^2^); 120.5 (Csp^2^); 115.3 (Csp^2^); 70.5 (Csp^3^); 69.1 (Csp^3^); 62.2 (Csp^3^); 57.8 (Csp^3^); 54.0 (Csp^3^); 53.5 (Csp^3^); 29.8 (Csp^3^). ESI‐MS *m/z* calculated for [C_46_H_53_N_6_O_4_]^+^: 753.41, found 753.42.

### 3.11 Oligonucleotides and Compounds Solutions

RP‐HPLC‐purified oligonucleotides were purchased as lyophilized products from Eurogentec (Liége, Belgium). The DNA sequences used in this study were Tel23 (d(AGGGTTAGGGTTAGGGTTAGGGT), Tel23‐FD (d(Dabcyl‐AGGGTTAGGGTTAGGGTTAGGGT‐6FAM), SCR‐F (d(GGATGTGAGTGTGAGTGTGAGG‐6FAM), and coSCR‐D (d(Dabcyl‐CCTCACACTCACACTCACATCC). Oligonucleotides were resuspended in milliQ water to obtain 100 µM stock solutions, which were stored at ‐20°C. Before analyses, samples were diluted in the required buffer, heated at 95°C for 7 min and led to slowly cool down at room temperature overnight. Finally, 150 mM KCl was added to promote G‐quadruplex folding. Double‐stranded DNA (scr‐dsDNA) was prepared by annealing an equimolar solution of the two complementary strands SCR‐F and coSCR‐D as above described.

All synthesized compounds were resuspended in DMSO to a final concentration of 4‐10 mM. Stock solutions were stored at ‐20°C until use.

### 3.12 Fluorescence Thermal Shift Assay (FTSA)

Experiments were performed in a Roche LightCycler 480 II, using an excitation source at 488 nm and recording the fluorescence emission at 520 nm. As target DNA, the G4‐forming telomeric sequence (Tel23‐FD, d(Dabcyl‐AGGGTTAGGGTTGGGTTAGGGT‐6FAM) and a double‐stranded DNA (scr‐dsDNA), both designed to localize a fluorophore (6‐FAM) and a quencher (Dabcyl) in proximity when folded. This setup allowed the quenching of the FAM fluorescence signal when the oligonucleotides were folded, while resulting in an increased fluorescence signal upon denaturation. Reaction mixtures (20 µl final volume) contained 0.5 µM DNA in 10 mM Li_3_(PO)_4_, 50 mM KCl, pH 7.5, and increasing concentrations of the tested compounds (0–80 µM). Samples were preliminarily heated and cooled in the 30‐90°C range at a rate of 0.1°C/s. Data were derived from subsequent heating and annealing steps run at a 1°C/min rate. Melting Temperatures (*T*_m_) were determined from the first derivatives of the melting profiles using the Roche LightCycler software, and Δ*T*_m_ was derived by subtracting the *T*_m_ value recorded in the presence of the ligand from the corresponding value in its absence. Each datapoint was repeated three times, and errors were ± 0.4°C.

### 3.13 UV‐Visible Spectroscopy

UV spectra were collected on a Jasco V‐730 spectrophotometer fitted with a Peltier thermostat‐regulated cell holder. All spectra were acquired in the 230‐700 nm range using a 1 cm pathlength cuvette and were corrected for the baseline. For each ligand, the molar extinction coefficient ε (M^−1^ cm^−1^) was calculated at the wavelength of maximal absorbance by monitoring the UV‐Vis spectra at increasing concentrations, in 5 mM TRIS, pH 7.5, at 25°C. When required, UV spectra were recorded in the 20‐95°C temperature range and in the presence of methanol.

### 3.14 Circular Dichroism Spectroscopy (CD)

CD spectra were recorded in 5 mM TRIS, 150 mM KCl, pH 7.5, on a Jasco J‐810 spectropolarimeter equipped with a Peltier temperature control system using a 10 mm path length cell. Spectra were acquired at room temperature, in the absence and presence of increasing concentrations of the selected ligands, using the following parameters: 100 nm/min scanning speed, 2 nm bandwidth, 0.5 nm data interval, 2 s response. Spectra were subtracted with the baseline and converted to mean residue ellipticity θ (Molar ellipticity), according to the following equation:

[θ] = deg × cm^2^ × dmol^−1^

### 3.15 ESI‐MS Binding Studies

DNA sequences were obtained from Sigma‐Aldrich (Milan, Italy). Samples were heat‐denatured and folded in 150 mM ammonium acetate before incubation with the ligands. Stock solutions of the compounds were prepared in methanol. The final concentration of the oligonucleotide was 5 µM in 150 mM ammonium acetate, with a 10:1 compound/oligo ratio. Samples were acquired after an equilibration time of 30 min and methanol was added to the samples to obtain a stable ESI signal. Mass spectra were recorded by direct infusion ESI on a Thermo Fisher Scientific (Waltham, MA) LCQ Fleet ion trap mass spectrometer. The instrument was set in negative ionization mode with a 3.4 kV capillary voltage, 120°C capillary temperature and a flow rate of 5 µL/min. CID experiments were performed on the complexes by isolating the precursor ion in the trap. The fragmentation was promoted by increasing the “normalized collision energy” (NCE) parameter. For data processing, Qual Browser Thermo Xcalibur 4.0.27.13 software was used. Exact mass for the Tel23: 7270.774 Da; for dsDNA (d(ACTATTTACGTATAATGA) paired to d(TCATTATACGTAAATAGT): 10987.922 Da.

The following equation was used to calculate BA, in which “I” is the intensity of the peak of interest in the mass spectrum:

$$BA=\frac{I_{\left(DNA-ligand\;complex\right)}}{I_{\left(DNA-ligand\;complex\right)}+I_{\left(DNA\right)}}$$

Based on these data, selectivity of the ligands can be calculated as the ratio between the BA_(G4‐ligand)_ and BA_(dsDNA‐ligand)_.

In CID studies, E_COM_^50%^ can be calculated based on the relative intensity of the adduct and the dissociation products in the MS spectra recorded at increasing collision energies. The proceeding reaction is measured by MS/MS and consists in the dissociation of AQA3 from the complex as reported in the following [26].

$${\left[ligand+GQ\right]}^{z-}\to \;{\left[GQ\right]}^{z-}+{\left[ligand\right]}^{z-}$$

Thus, the relative intensities of the complex and the fragmentation products can be plotted to obtain a dissociation curve by using the following equation, to calculate the E_COM_^50%^ of the complex:

$$E_{COM}=\frac{I_{complex}}{I_{complex}+I_{dissociation\;products}}$$

### 3.16 Computational Studies

Atomic coordinate files were retrieved from the RCSB Protein Data Bank (PDB, www.rcsb.org): the selected 3D structures are 1KF1 [31], 2JPZ [32], and 143D [33], which are all telomeric sequences formed by the d[G_3_(T_2_AG_3_)_3_] arrangement with parallel, hybrid‐2, and antiparallel topologies. Sequence preparation and docking were carried out in accordance with a previous work [17, 25, 34], and the head and tail caps were removed from the structures. Docking studies were performed using Glide [35, 36]. A blind SP docking approach was applied to investigate the interaction of AQAp and AQA3 toward the DNA arrangements. The search grid was set according to the following parameters: x = 23.99, y = ‐2.09, z = ‐8.74; size: 45 x 45 x 45 Å. In the protocol, at most two poses per ligand that underwent a postdocking minimization step were set. The best‐scoring pose was considered for further analysis. Schrödinger Maestro was used to produce the artworks (Schrödinger Release 2025‐1. Maestro‐Desmond Interoperability Tools, Schrödinger, New York, NY, USA 2021).

MD simulations were performed using the GPU‐accelerated Desmond tool of Schrödinger LLC (Schrödinger Release 2025‐1: Desmond Molecular Dynamics System, D.E. Shaw Research, New York, NY, USA, 2021. Maestro‐Desmond Interoperability Tools, Schrödinger, New York, NY, USA 2021). The best G4‐ligand complex obtained in the molecular docking phase was used to prepare a solvated system to perform MD, and was prepared according to Islam et al. [37] By using System Builder [38], the complex was solvated using the explicit TIP4Pew water model. Periodic boundary conditions were set with an orthorhombic box shape of 13 × 13 × 13 Å. During ion placement, K^+^ ions were used to neutralize the system; a concentration of 0.15 M of KCl was set. A run of 100 ns was performed under the OPLS4 force field with the temperature set at 300K and pressure at 1.0 bar. Further, the Martyna−Tuckerman−Klein chain coupling scheme with anisotropic coupling constant of 2.0 ps for the pressure control and the Nosé‐Hoover chain coupling scheme for the temperature control (NPT ensemble) were used. The cutoff radius in the Coulomb interactions was 9.0 Å. A RESPA integrator was set with a time step of 2.0, 2.0, and 6.0 fs, respectively, for bonded interactions and near and far interactions. Trajectories were saved at 100 ps intervals for analysis. Then, interactions, RMSD, and RMSF of ligands/G4 complex were checked. Artworks were made with Schrodinger Maestro.

### 3.17 Cell Viability Assay

The human gastric carcinoma cell line HGC‐27 (ECACC, catalogue no. 94042256) was cultured in Eagle's minimal essential medium (EMEM), supplemented with 10% fetal bovine serum (FBS) and 1% penicillin/streptomycin (all from Merck KGaA, Darmstadt, Germany). Cells were maintained at 37°C in a humidified 5% CO_2_ atmosphere.

Cell viability was assessed using PrestoBlue reagent (Invitrogen) according to the manufacturer's protocol. Briefly, cells were seeded in 96‐well plates at densities of 4000 or 1000 cells/well for the 24 and 72 h treatment, respectively. After overnight incubation, the culture medium was replaced with fresh medium containing increasing concentrations of tested compound, with a final vehicle concentration of 0.5% DMSO. Cells treated with vehicle alone were used as control. After treatment, the medium was removed and 100 µl of PrestoBlue reagent diluted 1:10 in culture medium was added to each well. Cells were incubated for 3 h at 37°C, protected from light. Fluorescence intensity was measured using a Spark Multimode Microplate Reader (Tecan) with excitation at 560 nm and emission at 590 nm. After background subtraction, fluorescence values were normalized by defining untreated control cells as 100%.

## 4 Conclusion

In the present study, we synthesized and characterized the activity of a series of anthraquinone‐based ligands by exploiting different synergistic techniques to assess the potential interactions of these molecules with a telomeric G4‐forming sequence. Starting from the design of the most promising functional unit, the derivative AQAp gave us promising outcomes in terms of G4 recognition over duplex DNA, as confirmed by biophysical studies. Moreover, AQAp resulted cytotoxic in a human gastric cancer cell line.

Based on the output derived from these experiments, we initiated an optimistic design of bivalent G4 binders, consisting of two conserved aromatic portions spaced by a flexible linker. In our primary intention, this concept would serve to exert a double stacking effect on the external tetrads of the G4, thus enhancing the affinity and selectivity of the compound. Although preliminary ESI‐MS and computational studies enable us to achieve favorable results, we demonstrated that the flexibility of the linker impacts dramatically on the activity of the ligand. Specifically, a highly flexible spacer appeared to promote the stacking of the connected aromatic cores, making them unavailable to interact with the G‐quadruplex tetrads. Nevertheless, this approach represents a promising strategy to target G4s selectively. By regulating the nature of the linker and exploiting its possible interaction with grooves or the backbone, we speculate that the activity of these ligands can be increased, thus producing a new class of highly selective G4 binders.

## Conflicts of Interest

The authors declare no conflict of interest.

## Supporting information




**Supporting File 1**: chem70465‐sup‐0001‐SuppMat.pdf

## Data Availability

The data that support the findings of this study are available in the supplementary material of this article.
