# Supplementary material for: Enhancing G‐Quadruplex Binding: Rational Design and Biophysical Evaluation of Dimeric Ligands
Source: Chemistry. 2025 Nov 23;31(72):e03128. doi: 10.1002/chem.202503128 (PMC12731523; doi:10.1002/chem.202503128)

# - Supporting information –

Enhancing G-Quadruplex Binding: Rational Design and Biophysical Evaluation of Dimeric Ligands

Matteo Giannangeli,†[a] Nicolò Dal Ponte,†[b] Margrate Anyanwu,[a] Martina Brigida Romanello,[a] Ernesto Mucenji,[b] Riccardo Rigo,[b] Giovanni Ribaudo,*[a] Claudia Sissi,*[b] and Alessandra Gianoncelli,[a]

[a] Dr. M. Giannangeli, Dr. M. Anyanwu, Dr. M.B. Romanello, Dr. G. Ribaudo, Prof. A. Gianoncelli
Department of Molecular and Translational Medicine, University of Brescia, Viale Europa 11, 25123 Brescia, Italy
E-mail: giovanni.ribaudo@unibs.it

[b] Dr. N. Dal Ponte, Dr. E. Mucenji, Dr. R. Rigo, Prof. C. Sissi
Department of Pharmaceutical and Pharmacological Sciences, University of Padova, Via Marzolo 5, 35131 Padova, Italy
E-mail: claudia.sissi@unipd.it

Table of content

Characterization of the synthesized compounds S2

ESI-MS binding studies S19

Computational studies S20

Evaluation of cytotoxicity S21

Characterization of the synthesized compounds

AQAp – ESI-MS

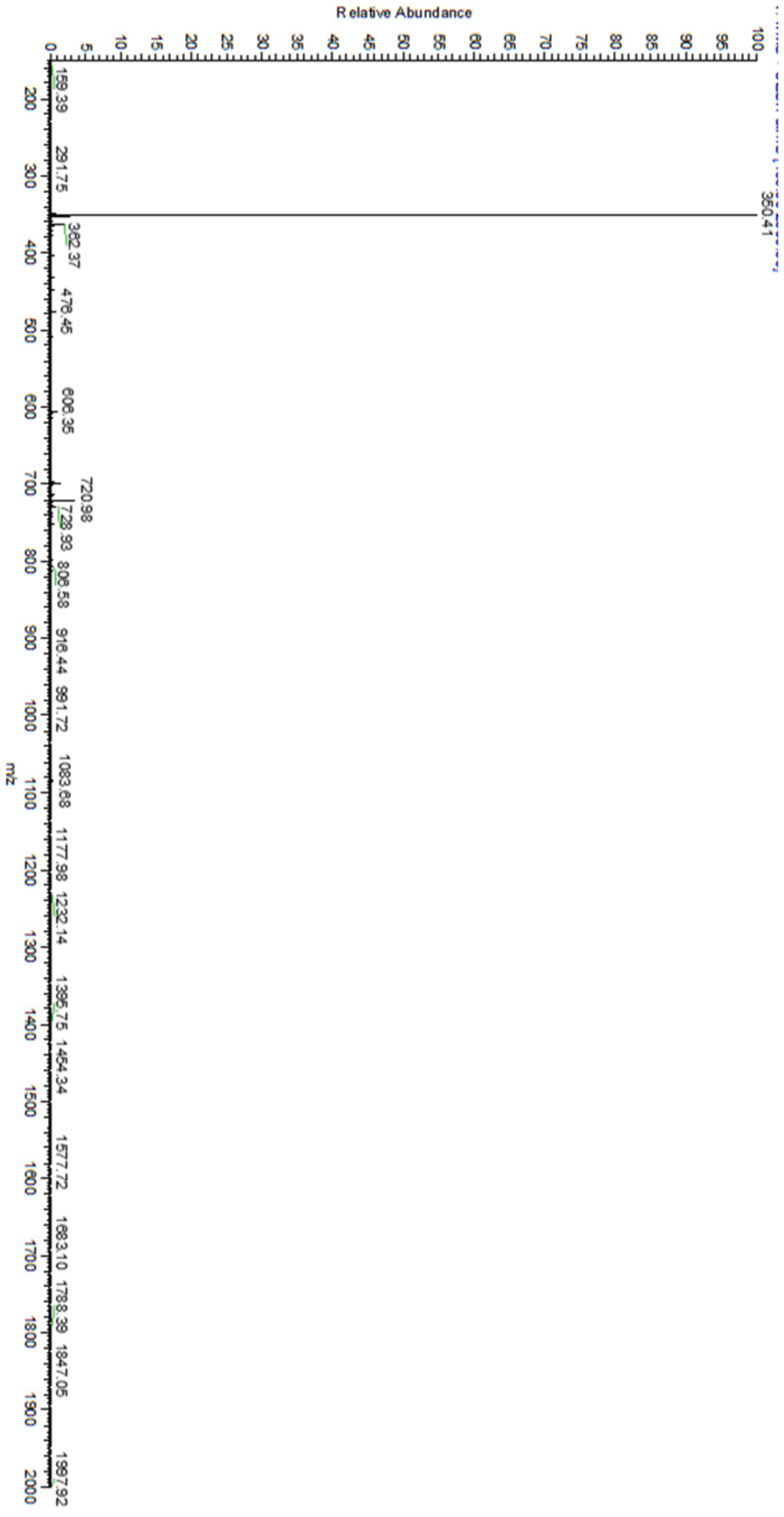

AQAp – ^{1}H NMR

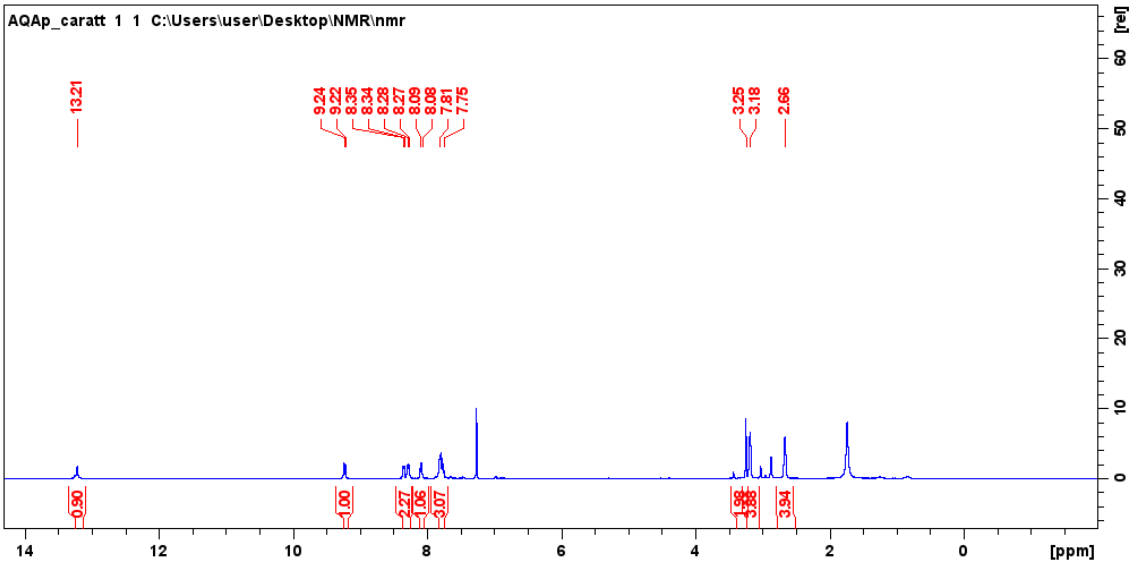

AQAp – ^{13}C NMR

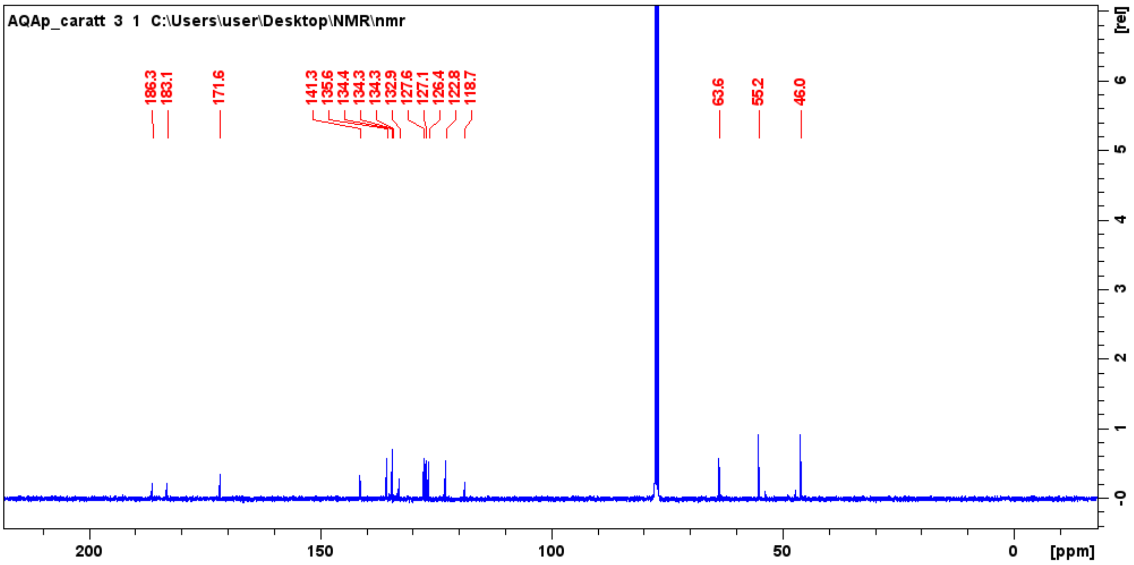

AQApBut – ESI-MS

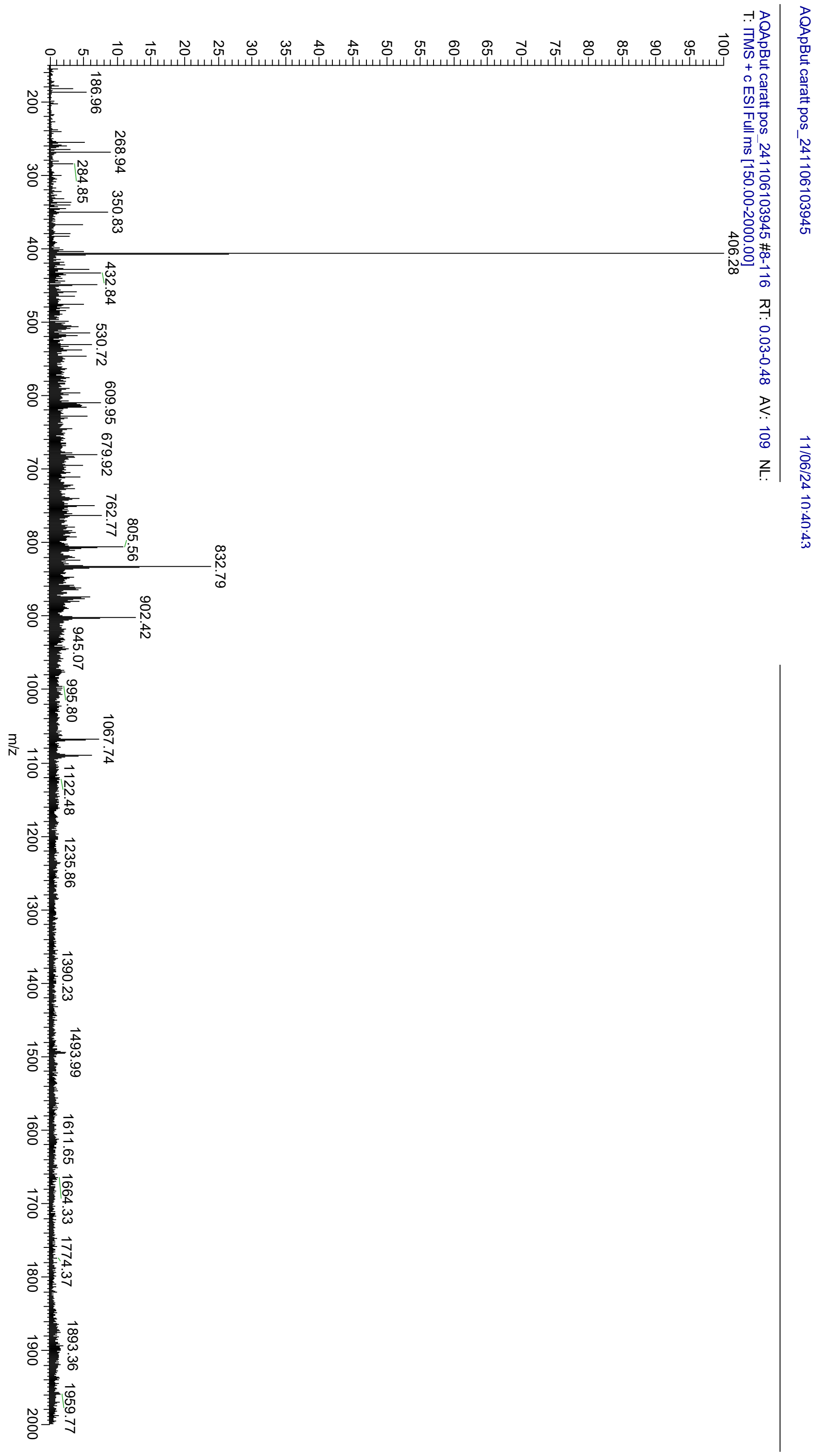

AQApBut – ^{1}H NMR

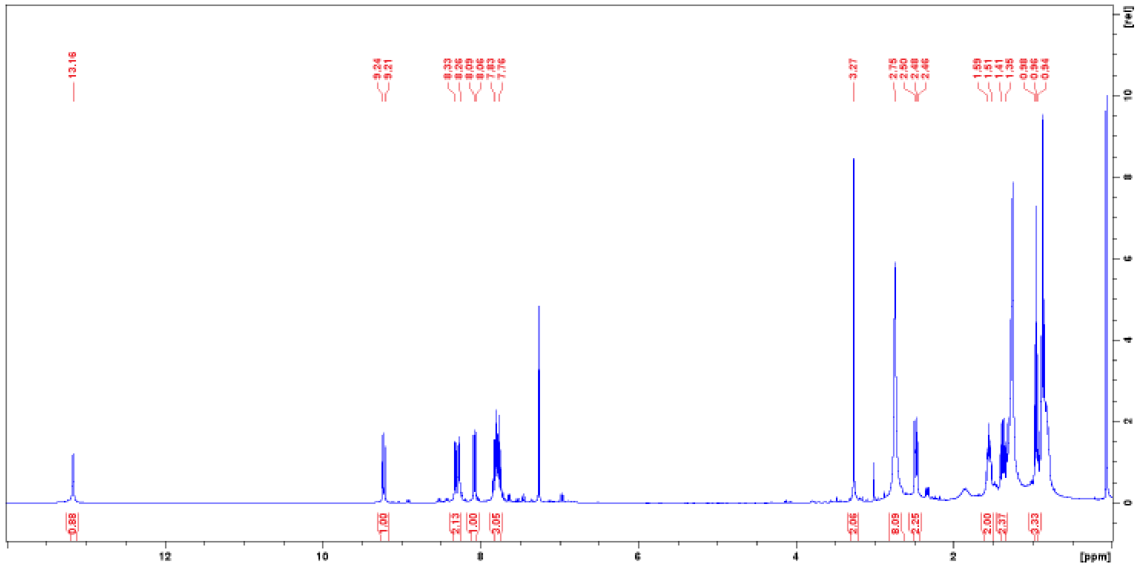

AQApBut – ^{13}C NMR

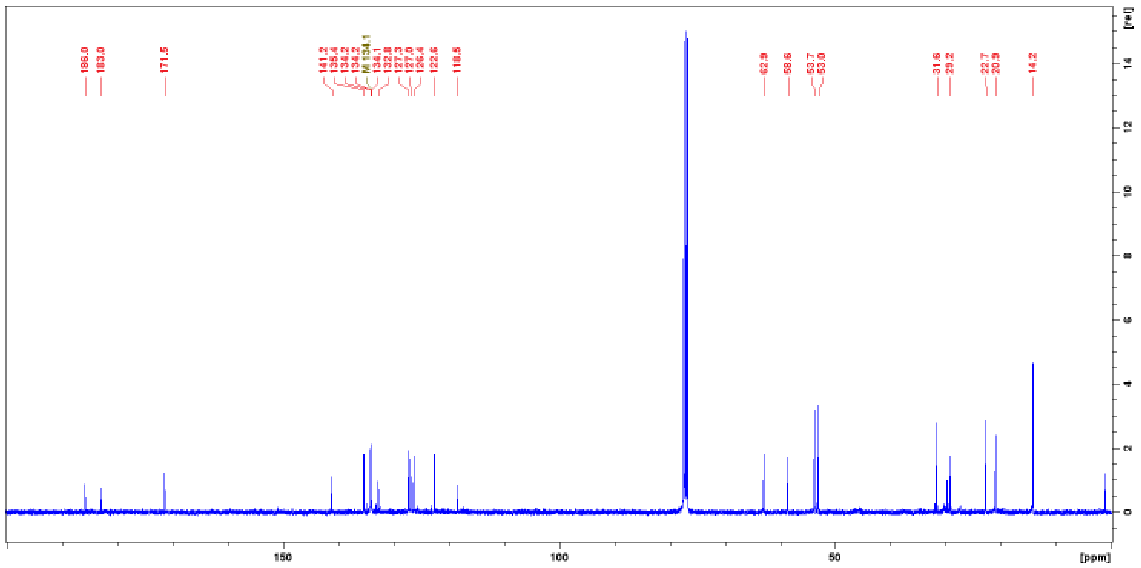

AQAep – ESI-MS

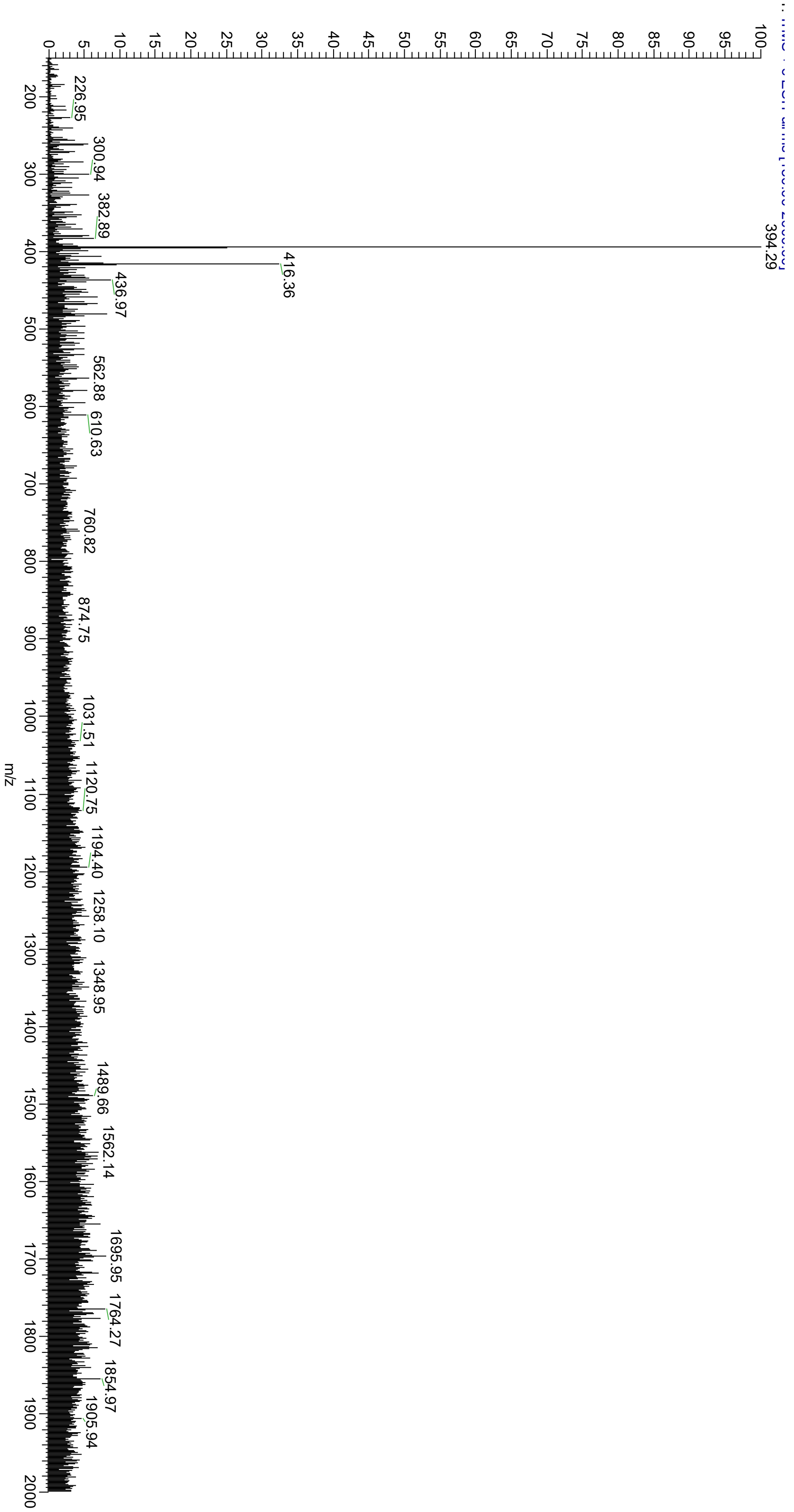
394.29
416.36
226.95
300.94
382.89
436.97
562.88
610.63
760.82
874.75
1031.51
1120.75
1194.40
1258.10
1348.95
1489.66
1562.14
1695.95
1764.27
1854.97
1905.94
m/z

AQAep – ^{1}H NMR

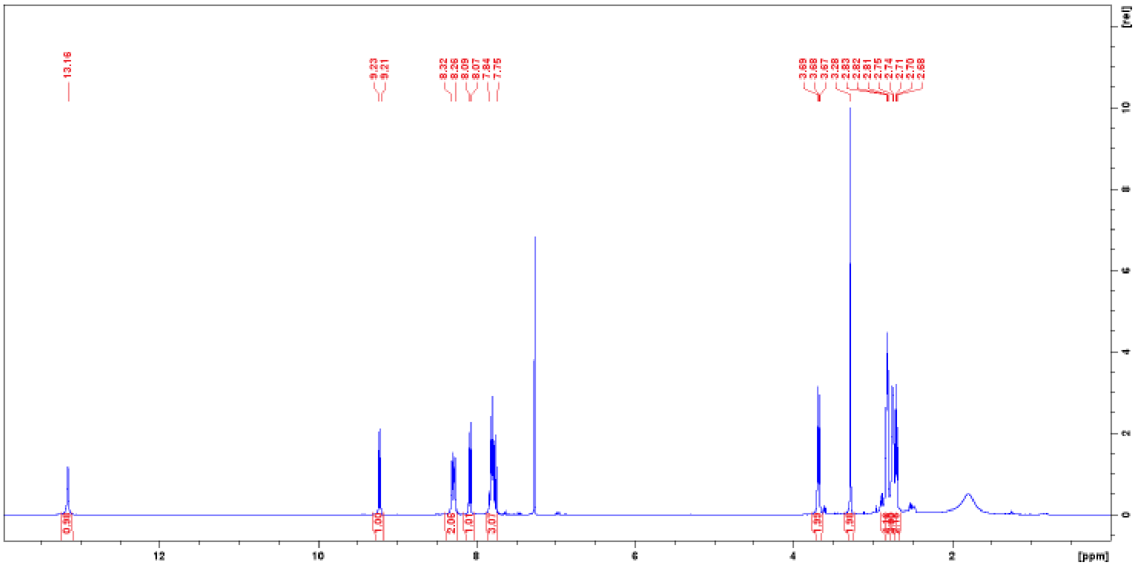

AQAep – ^{13}C NMR

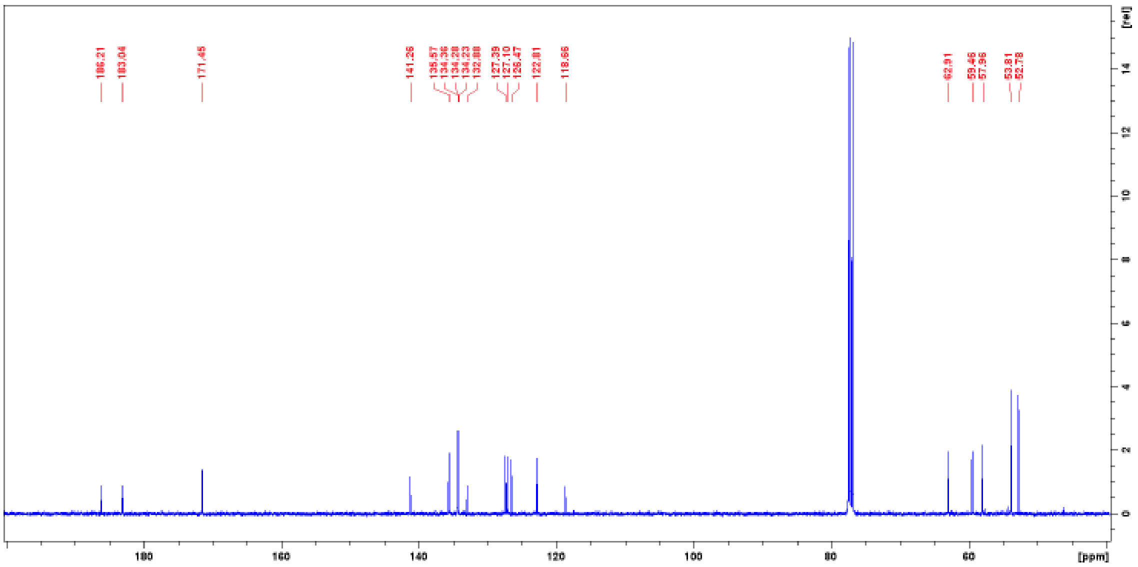

AQAbp – ESI-MS

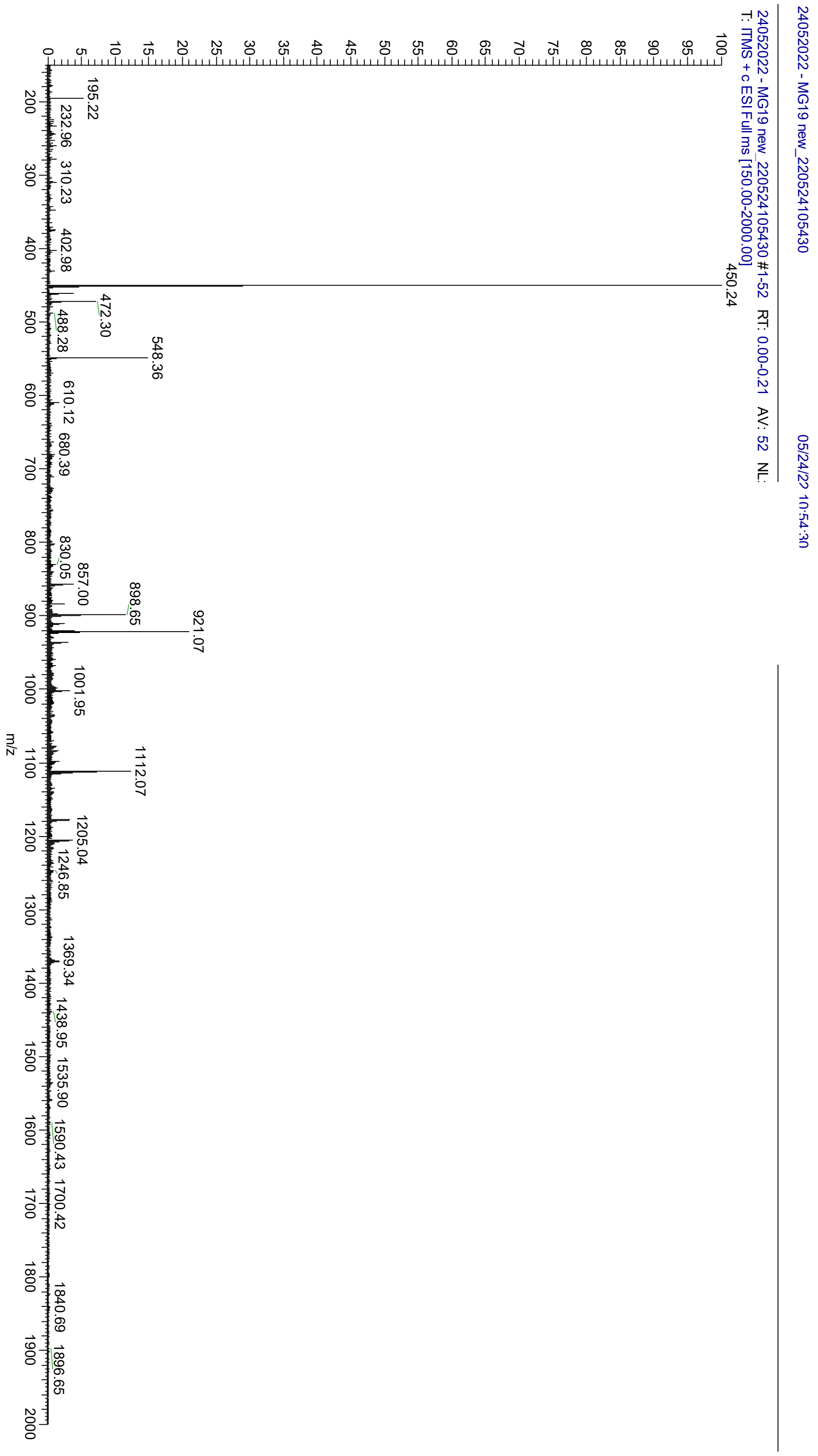
24052022 - MG19 new_220524105430
05/24/22 10:54:30
24052022 - MG19 new_220524105430 #1-52 RT: 0.00-0.21 AV: 52 NL:
T: ITMS + c ESI Full ms [150.00-2000.00]
450.24
548.36
921.07
898.65
1112.07
472.30
195.22
1205.04
857.00
1001.95
1369.34
232.96
310.23
402.98
488.28
610.12
680.39
830.05
1246.85
1438.95
1535.90
1590.43
1700.42
1840.69
1896.65
m/z

AQAbp - ^{1}H NMR

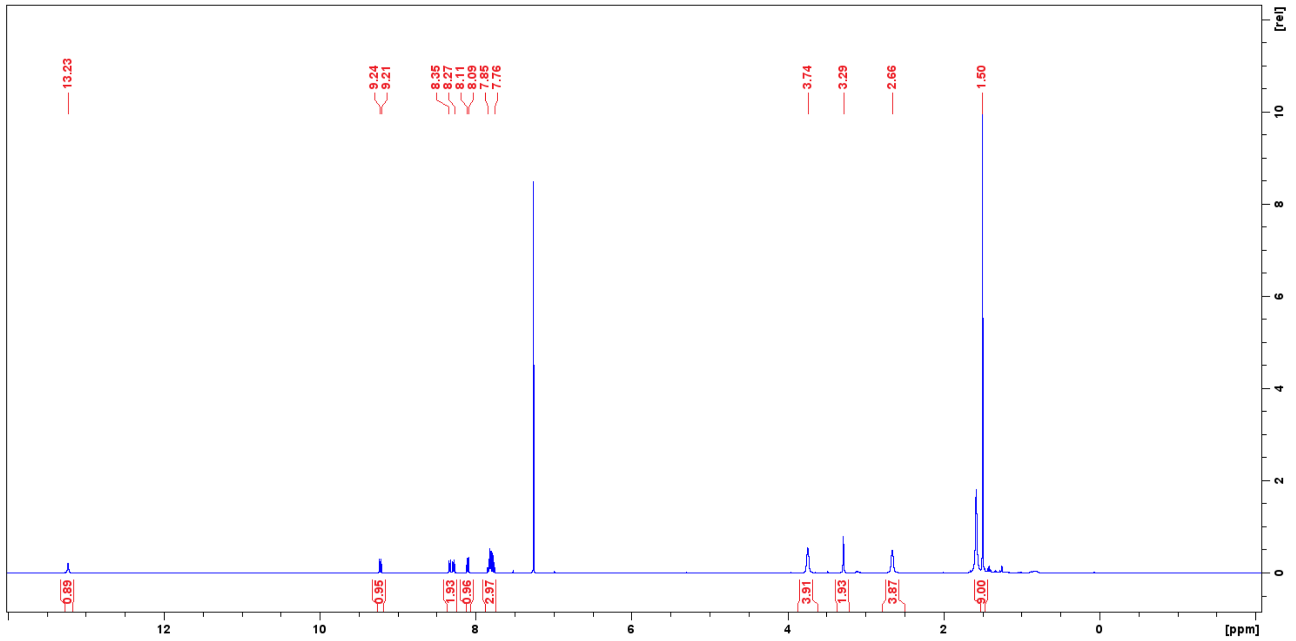

AQAbp – ^{13}C NMR

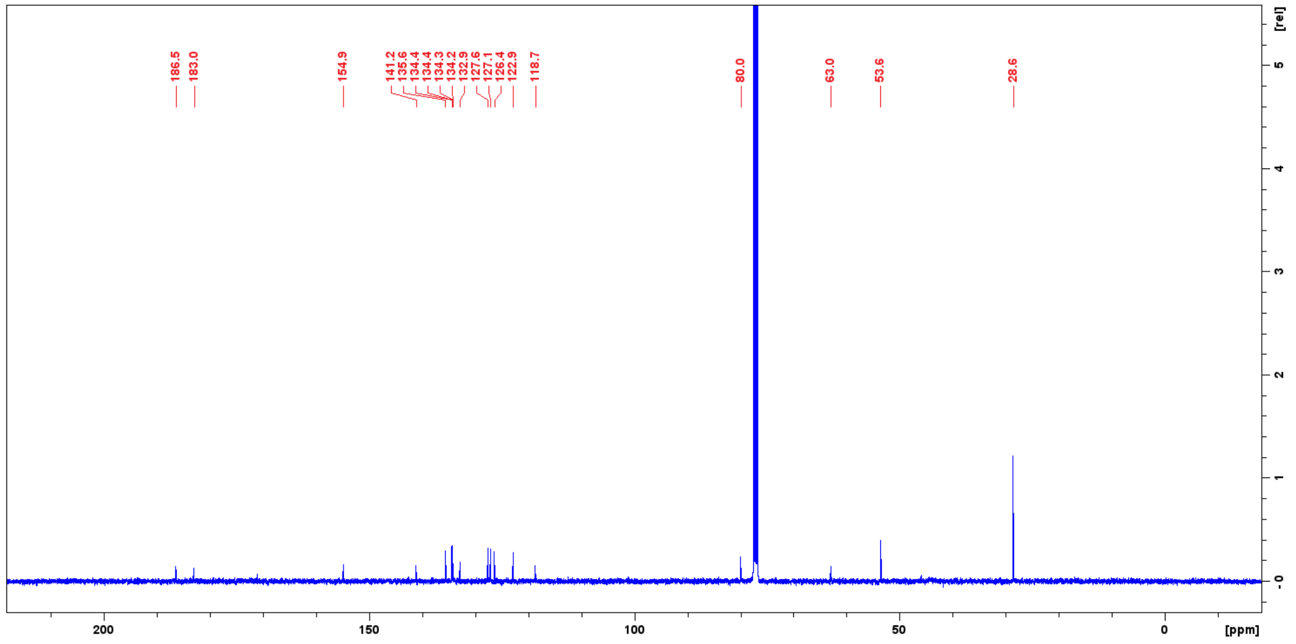

AQA3m – ESI-MS

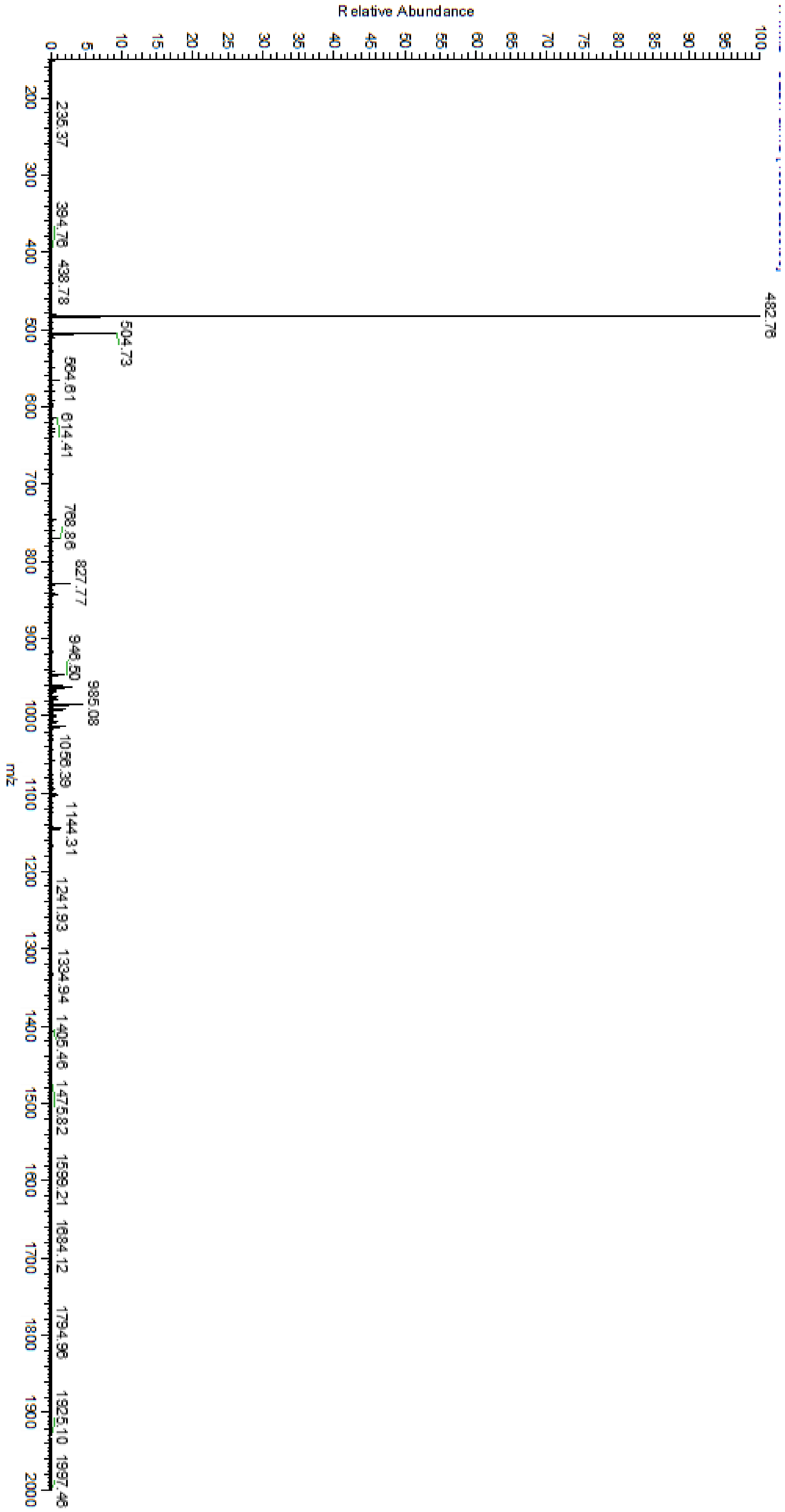
Relative Abundance
m/z
482.76
504.73
236.37
394.76
438.78
564.61
614.41
768.86
827.77
946.50
985.08
1056.39
1144.31
1241.93
1334.94
1405.46
1475.82
1589.21
1684.12
1794.96
1925.10
1997.46

AQA3m – ^{1}H NMR

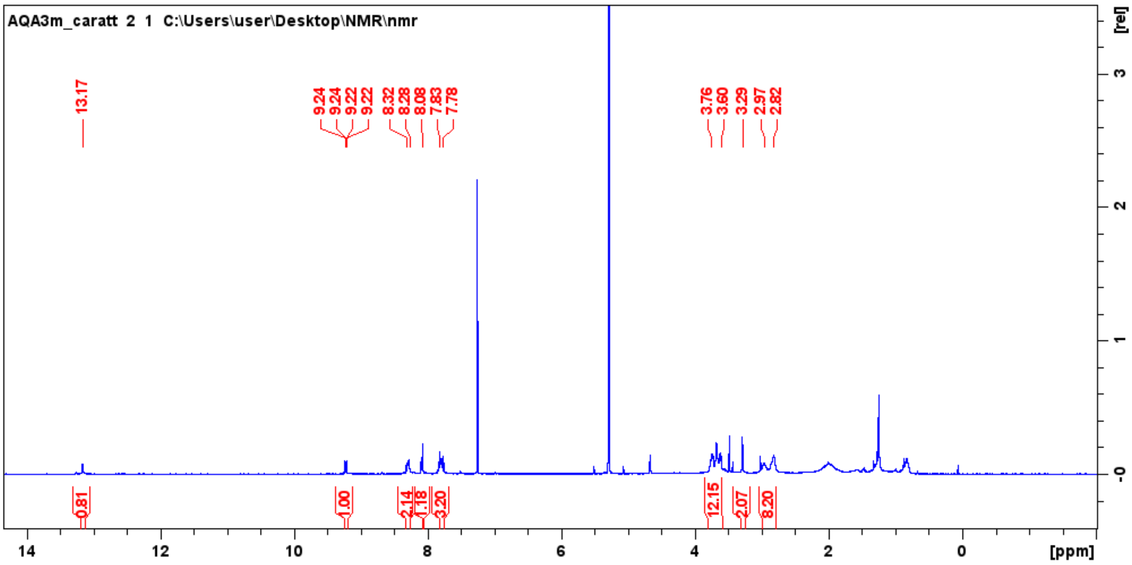

AQA3m – ^{13}C NMR

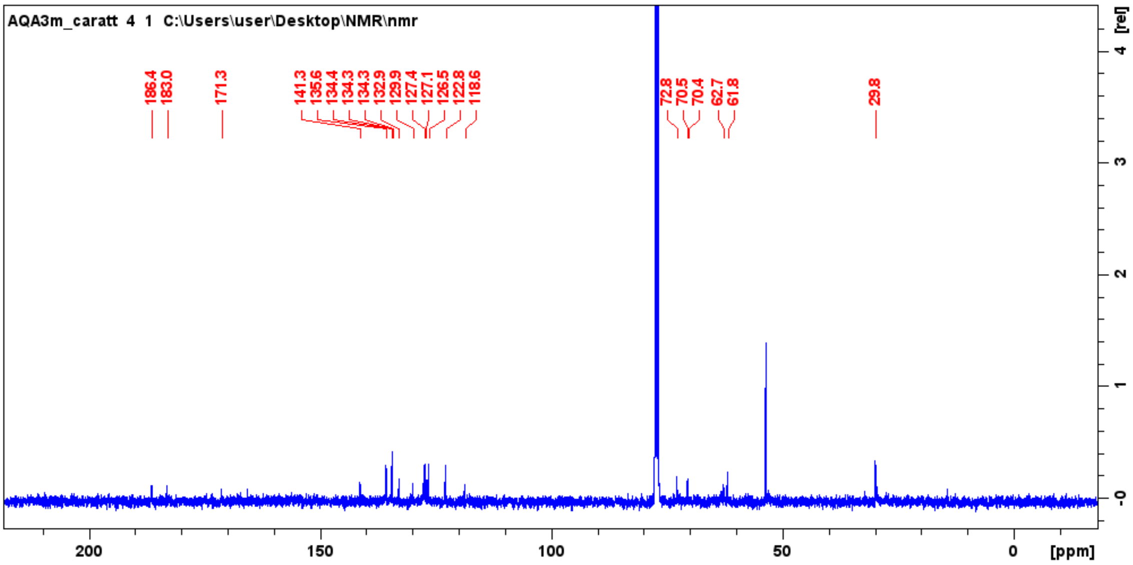

AQA3 – ESI-MS

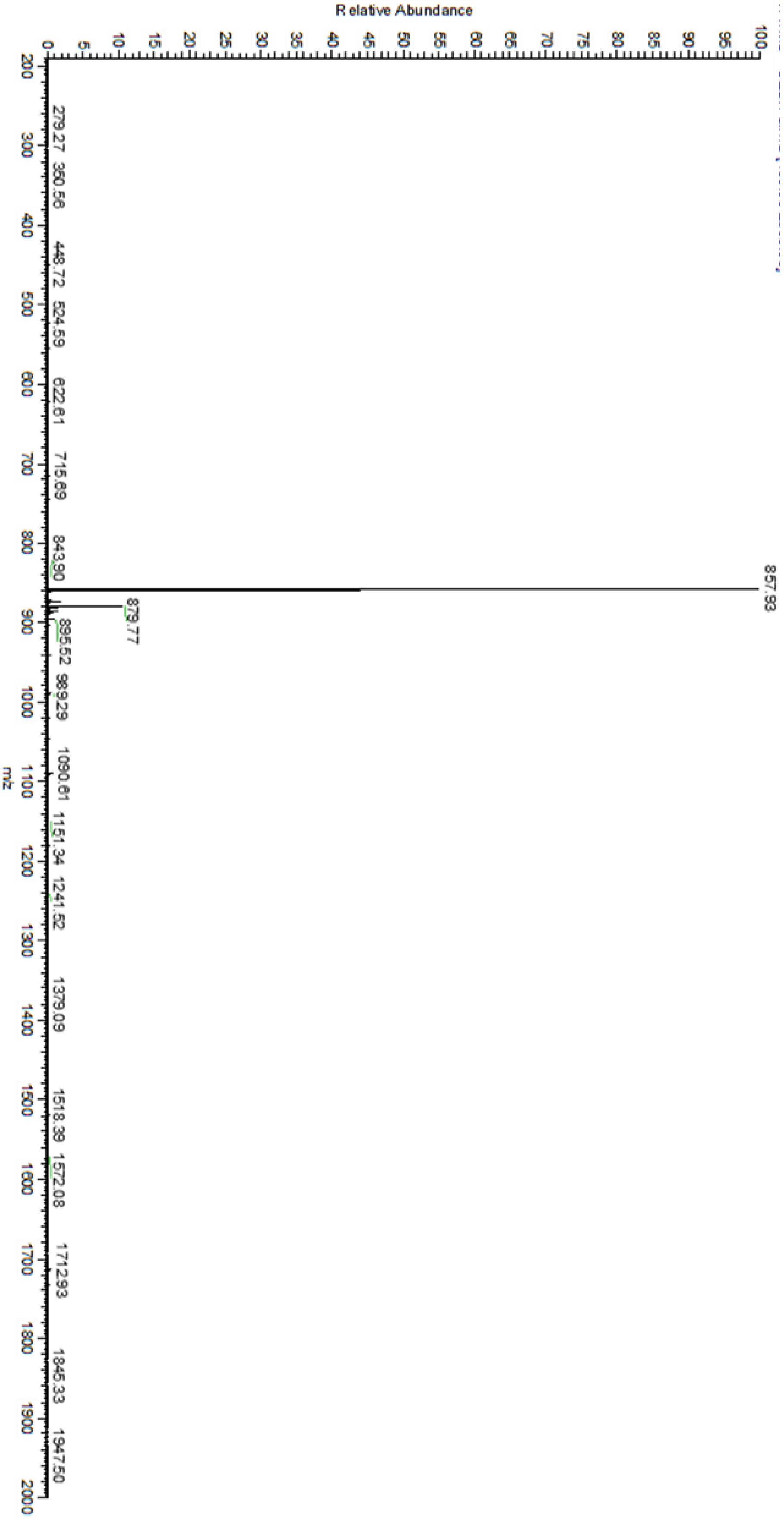

Relative Abundance
m/z
279.27
350.56
448.72
524.59
622.61
715.69
843.90
857.93
879.77
895.52
989.29
1090.61
1151.34
1241.52
1379.09
1518.39
1572.08
1712.93
1845.33
1947.50

AQA3 – ^{1}H-NMR

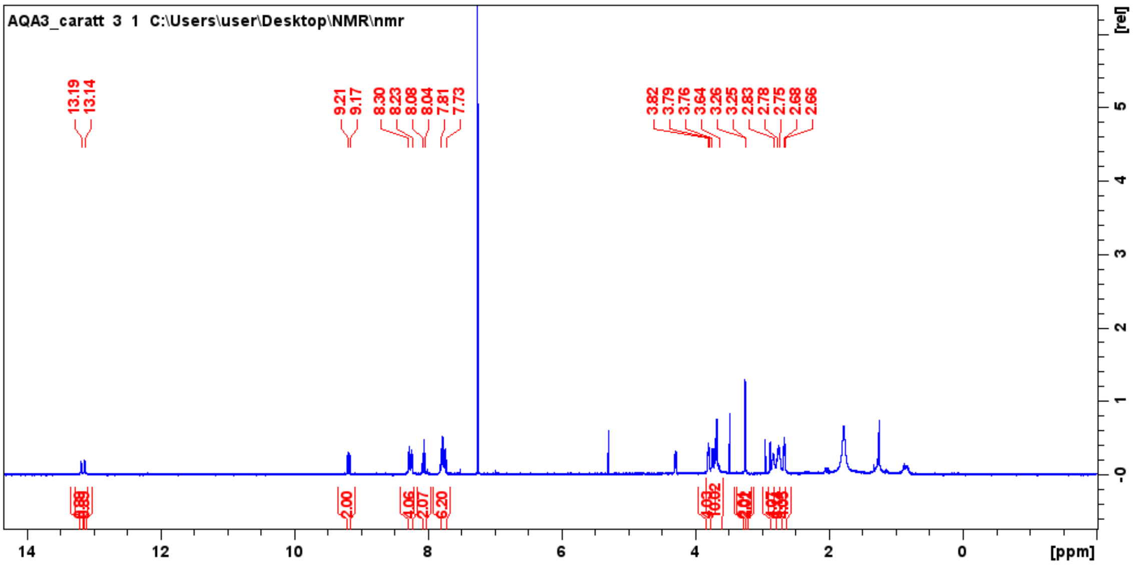

AQA3 – ^{13}C-NMR

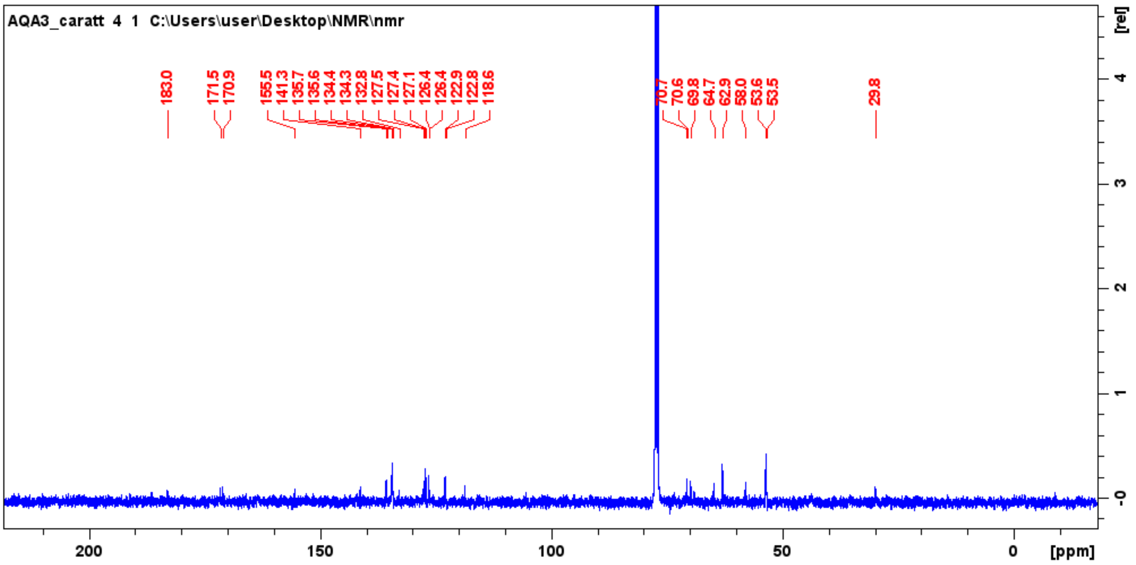

AQA3 – ^{1}H-NMR (variable temperature)

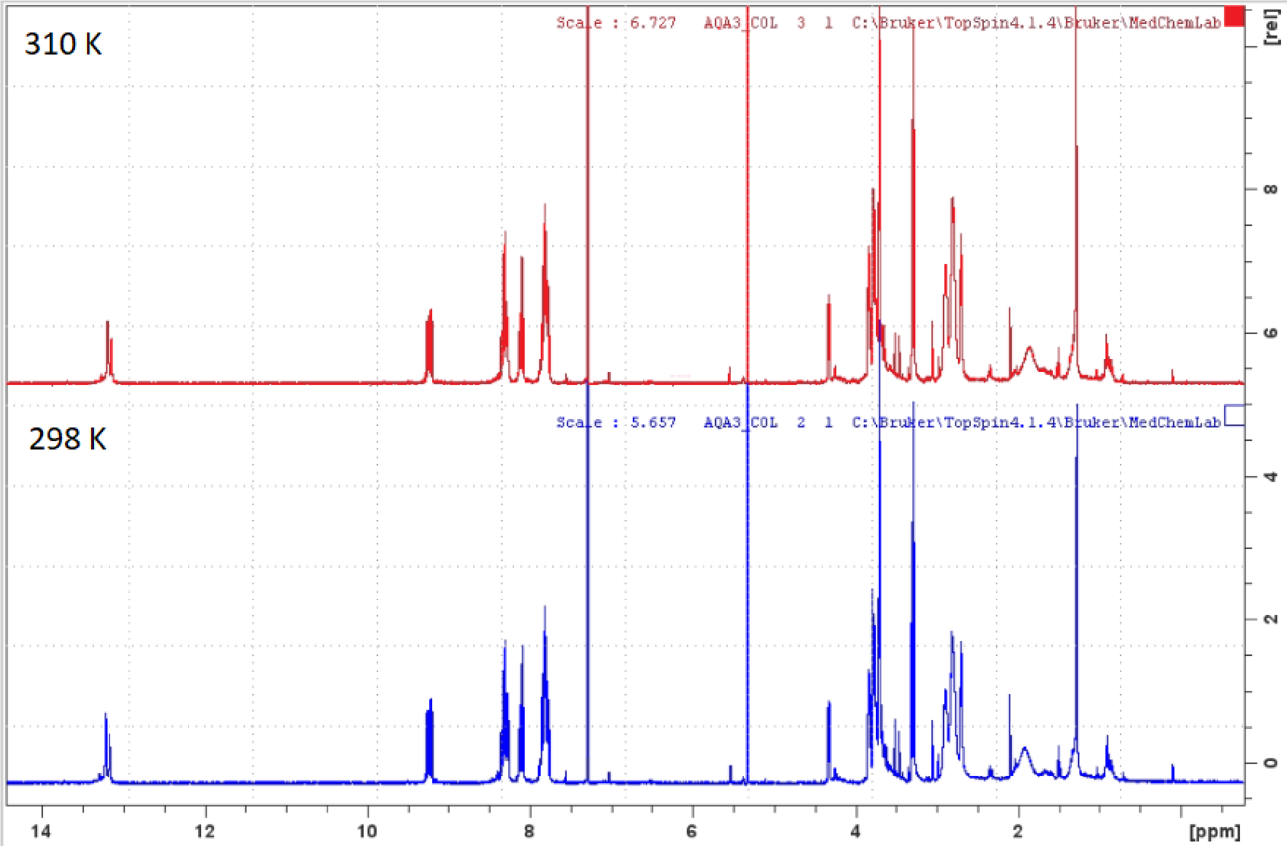

ANAp – ESI-MS

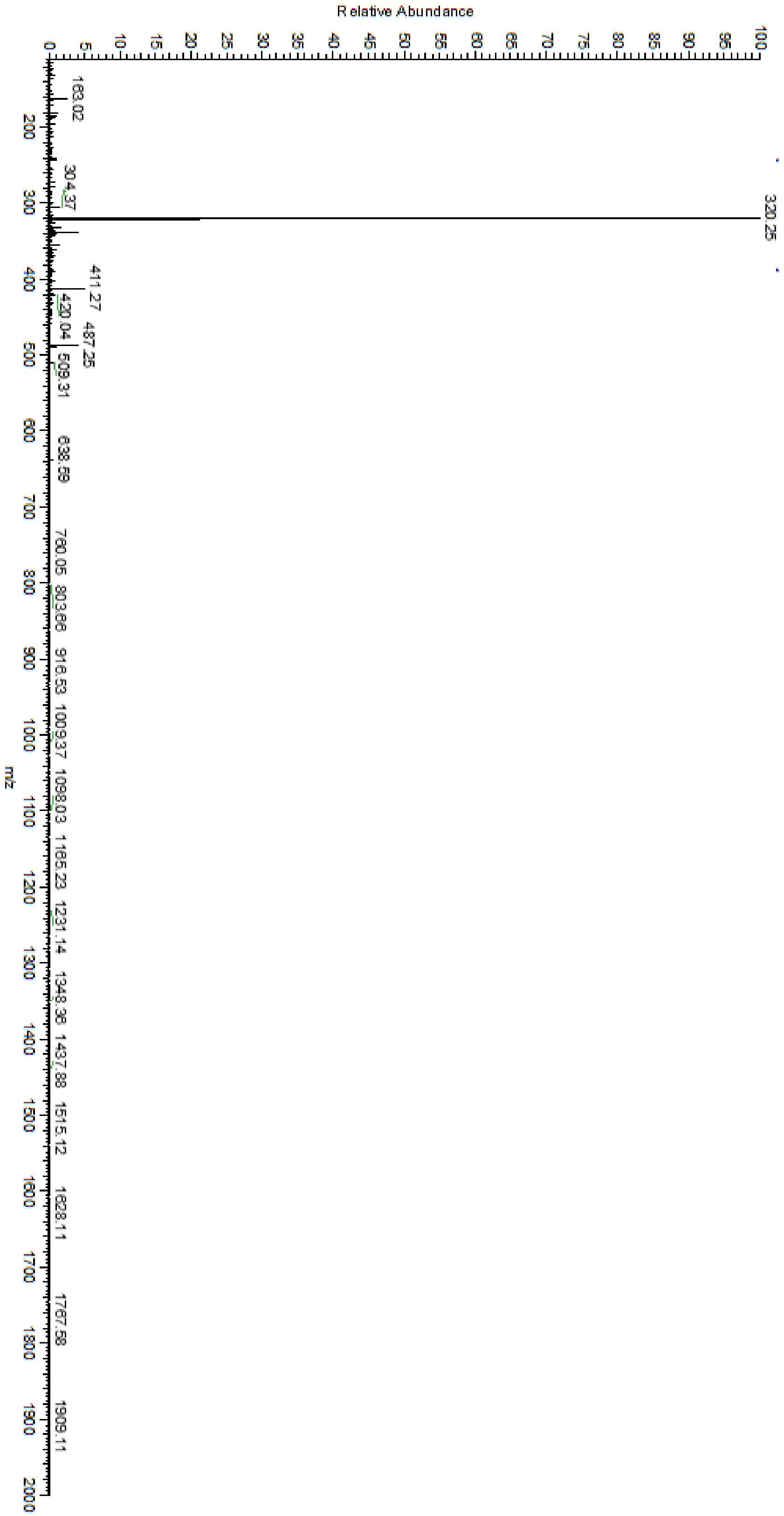
Relative Abundance
m/z
163.02
304.37
320.25
411.27
420.04
487.25
509.31
638.59
760.05
803.68
916.53
1009.37
1098.03
1165.23
1231.14
1348.36
1437.88
1515.12
1628.11
1767.58
1909.11

ANAp – ^{1}H NMR

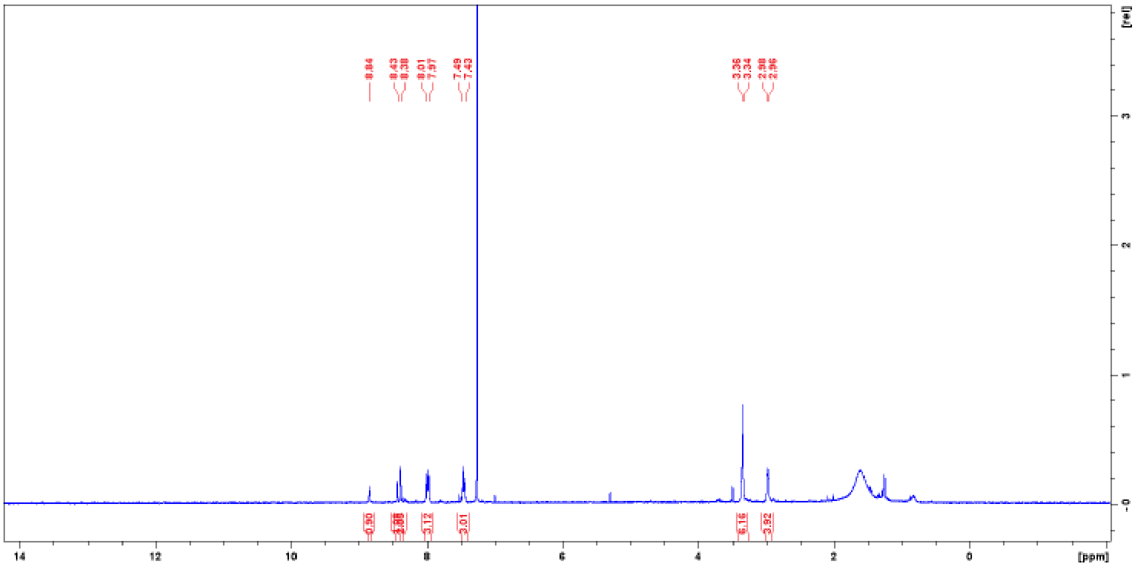

ANAp – ^{13}C NMR

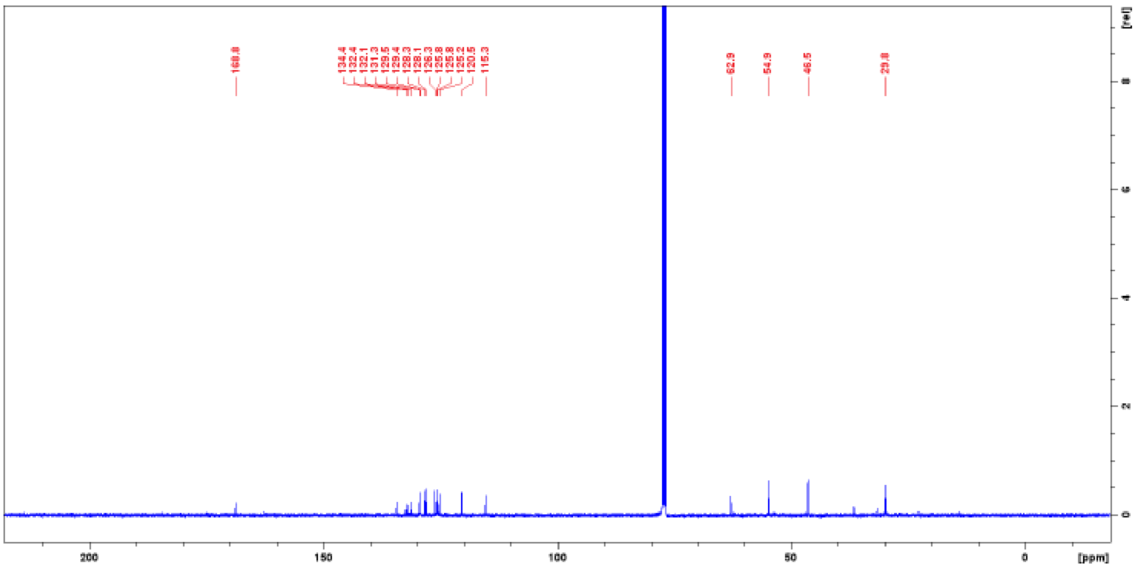

ANA3 – ESI-MS

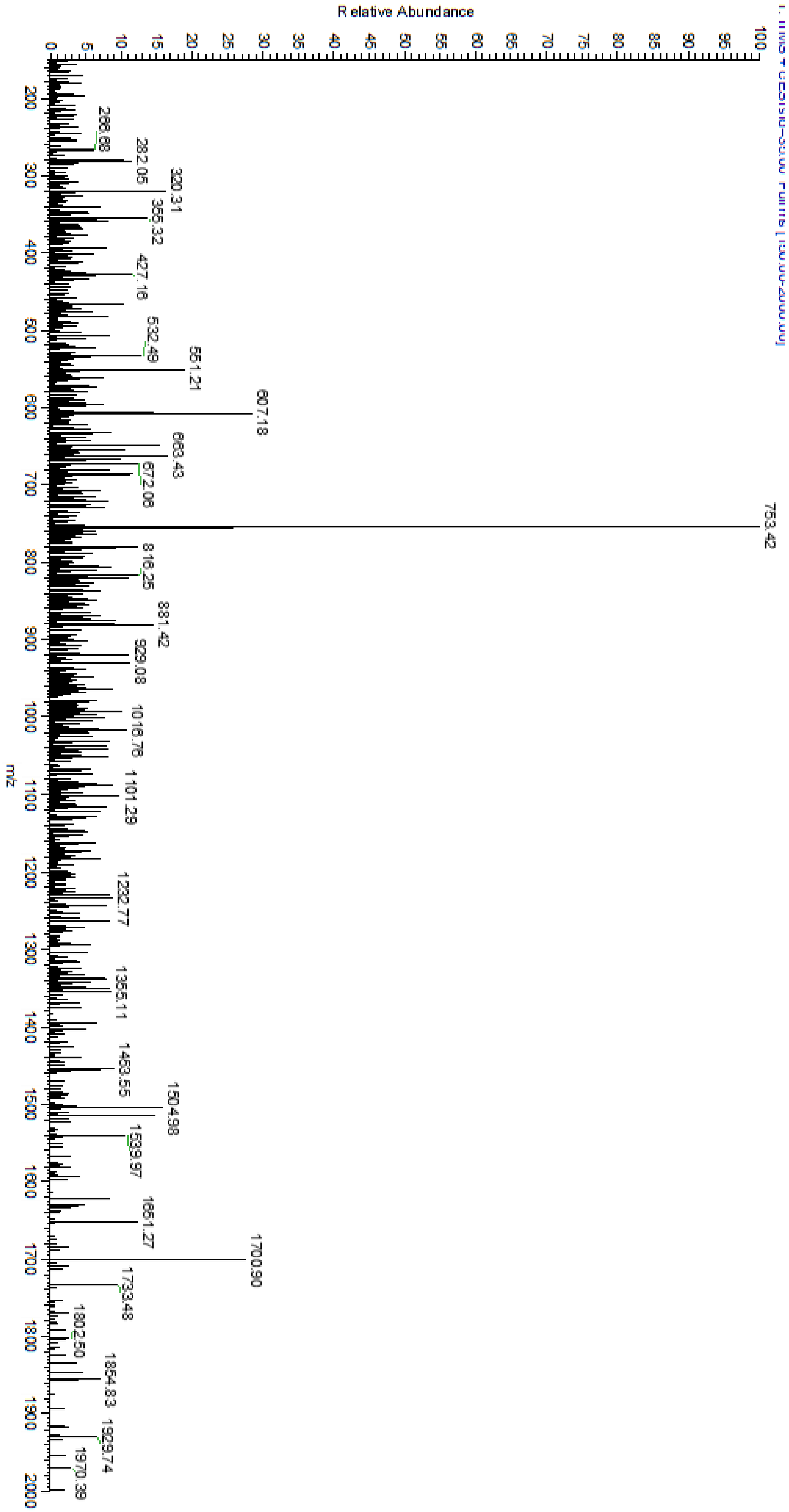
Relative Abundance
m/z
753.42
607.18
1700.90
551.21
1504.98
320.31
663.43
881.42
355.32
282.05
427.16
532.49
672.06
816.25
929.08
1016.78
1101.29
1232.77
1365.11
1463.55
1539.97
1651.27
1733.48
1854.83
1929.74
1802.50
1970.39
268.68

ANA3 – ^{1}H NMR

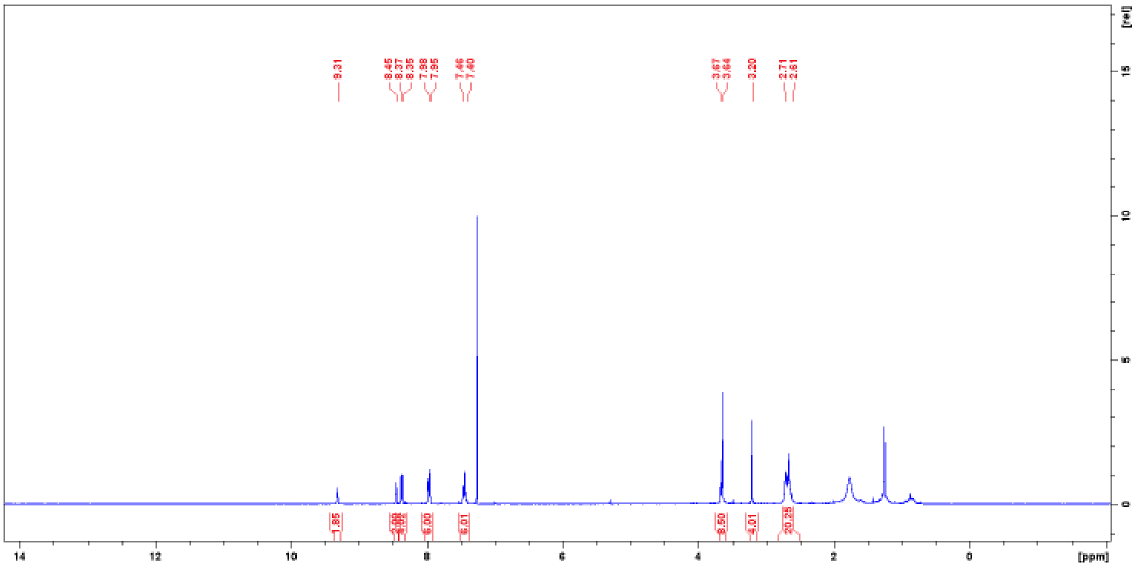

ANA3 – ^{13}C NMR

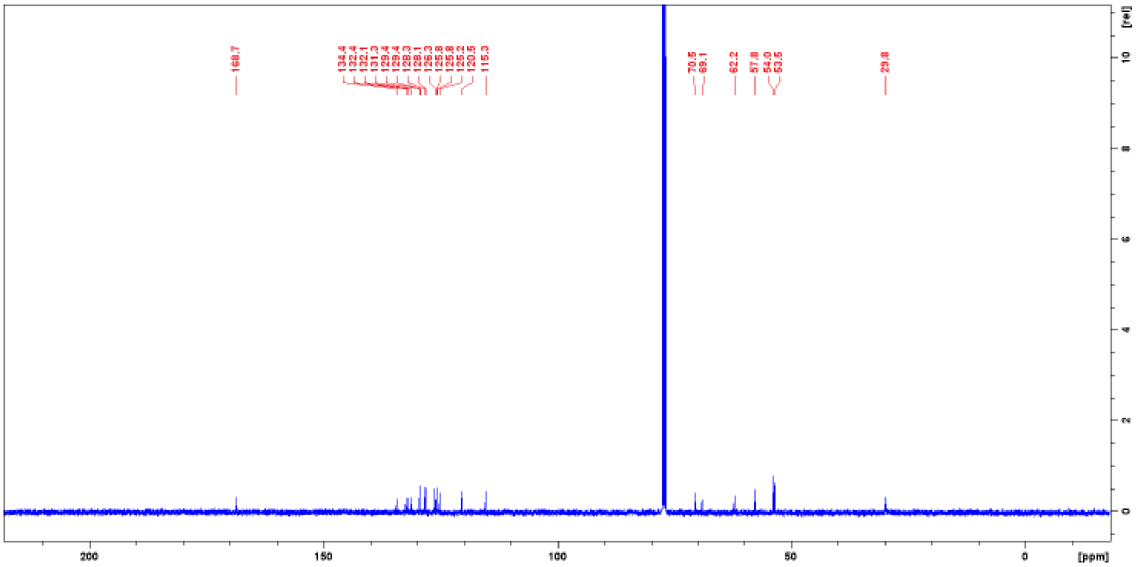

ESI-MS binding studies

AQAep and AQApBut binding studies

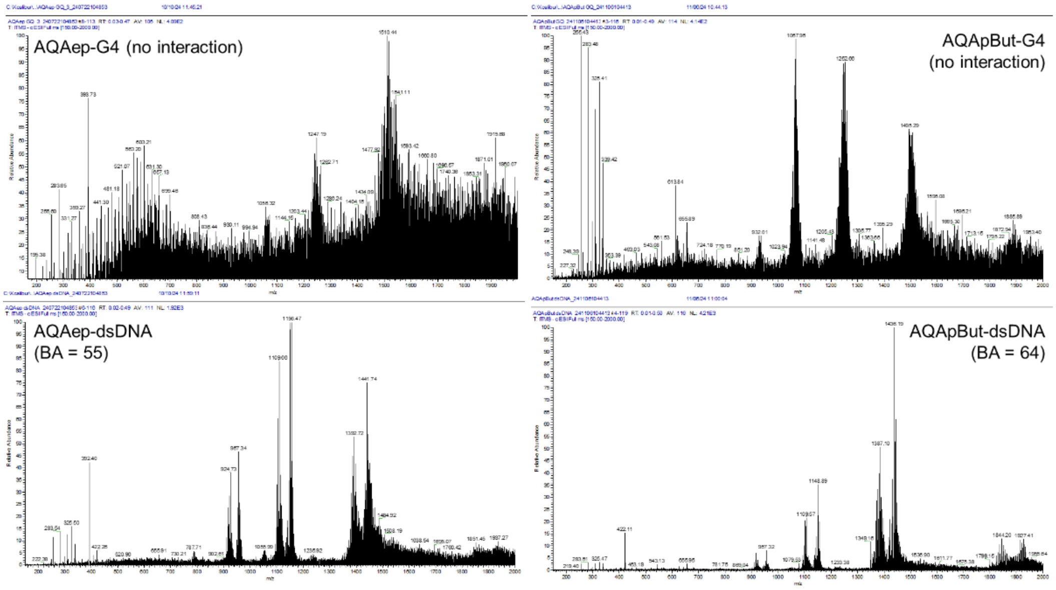

Collision-induced dissociation (CID) study for ANAp ($E_{COM}^{50\%}$ = 46.1 eV)

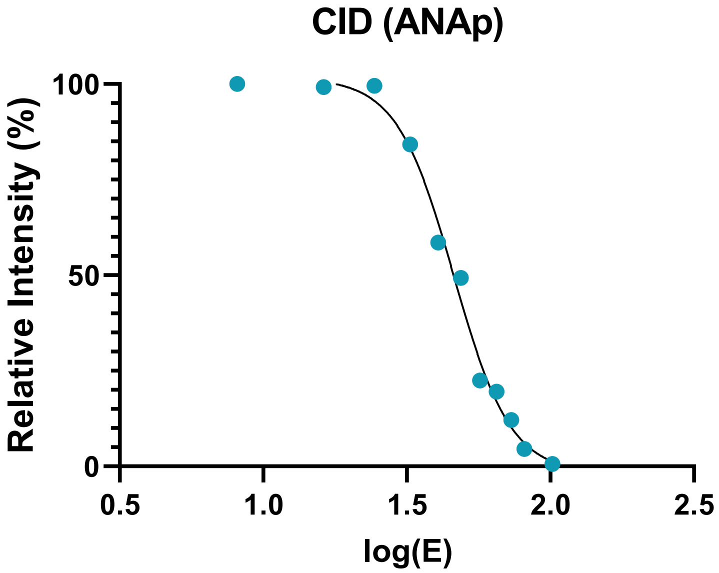

Computational studies

Clustered docking poses for AQA3 towards 1KF1 (**A**), 2JPZ (**B**), and 143D (**C**).

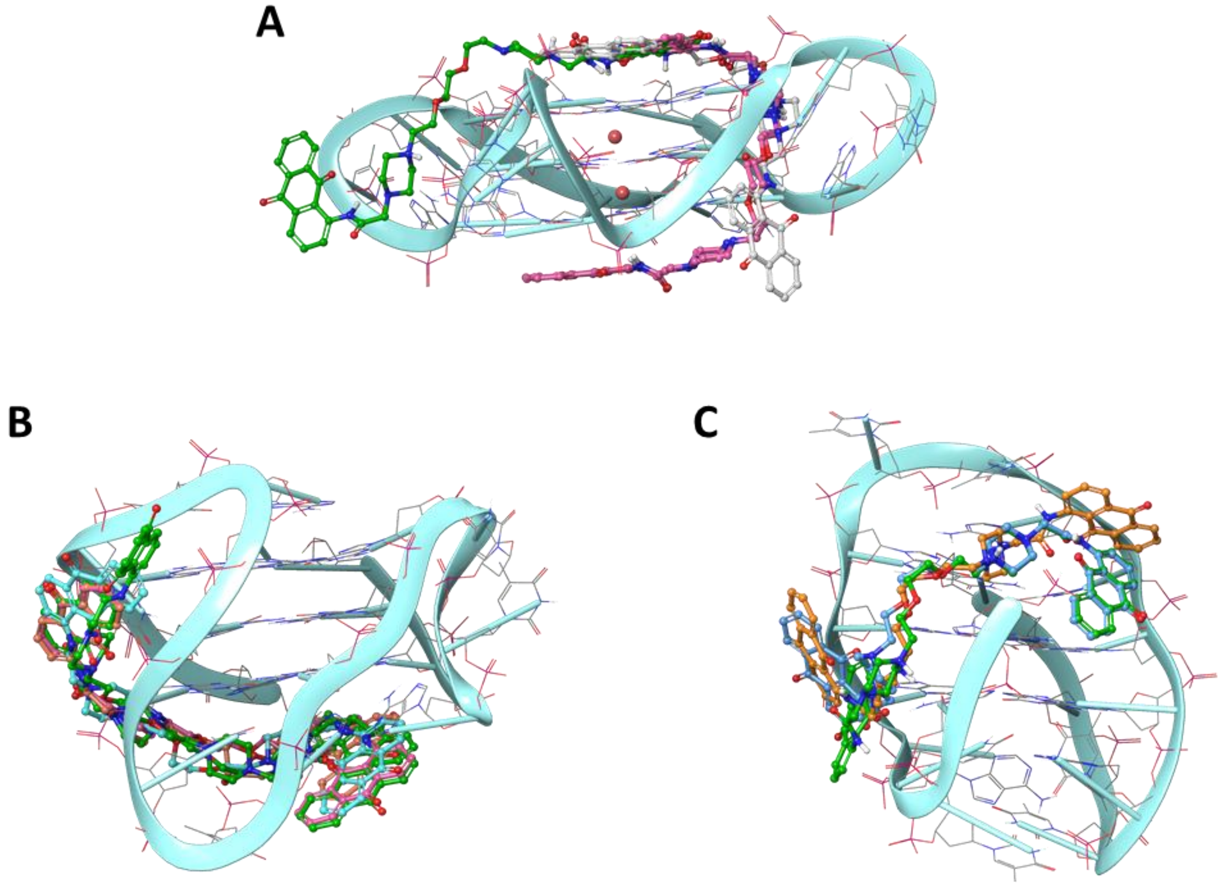

Interaction plot of complexes 1KF1/AQA3t (**A**), 1KF1/AQA3h (**B**), 1KF1/AQAp (**C**).

A

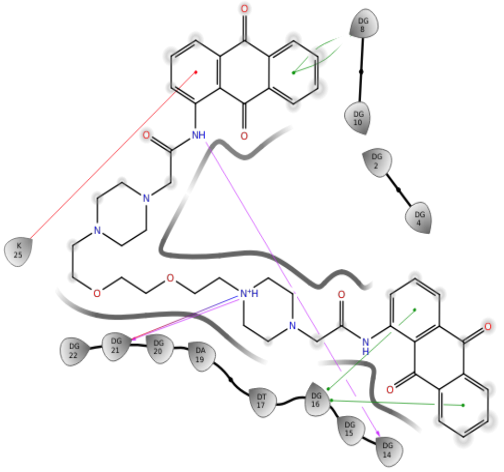

B

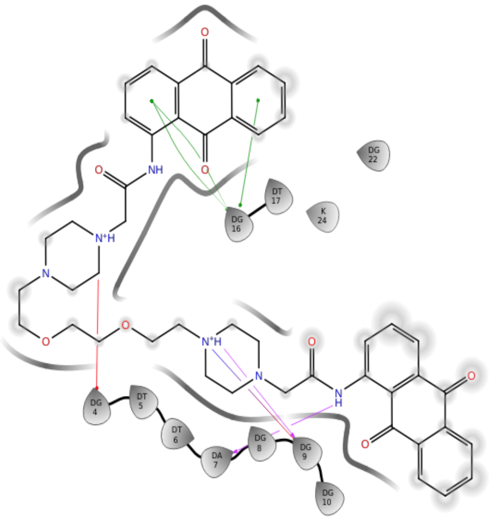

C

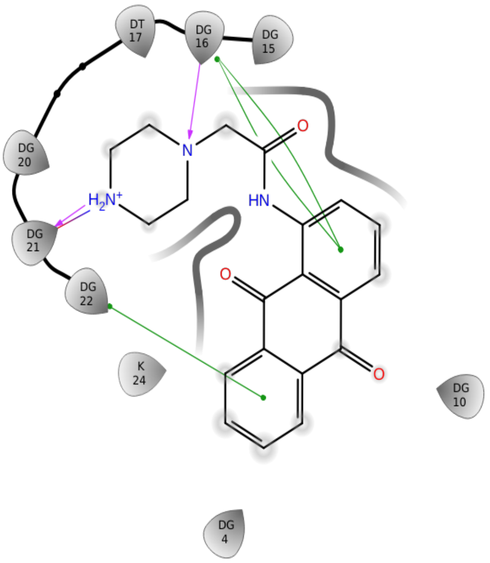

Evaluation of cytotoxicity

Plot of the % cell viability measured by the PrestoBlue assay on HGC-27 cell line after **A**) 24 h and **B**) 72 h with increasing concentration of AQAp and AQA3 (0−50 µM). The curve was fitted by using the dose−response equation; error bars represent the standard deviations derived from three independent experiments.

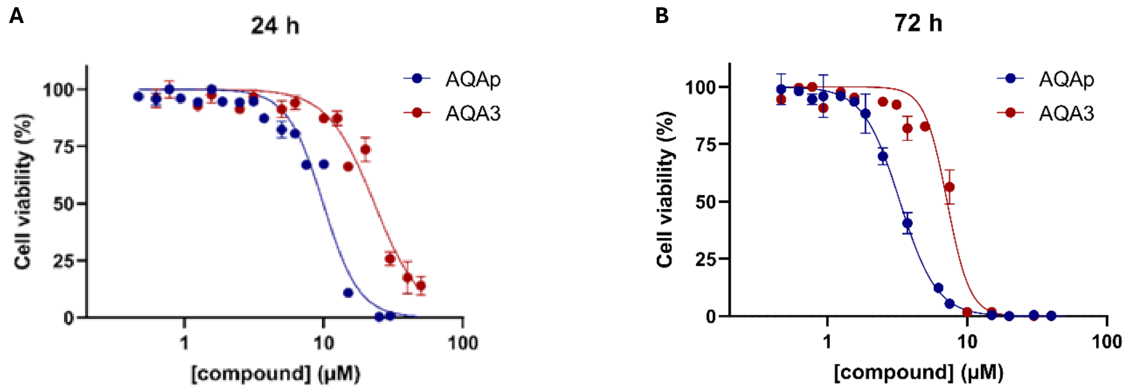

Supplement: Supplementary file 1 — Supporting File 1: chem70465‐sup‐0001‐SuppMat.pdf [file CHEM-31-e03128-s001.pdf]
